# Intraspecific trait variability facilitates tree species persistence along riparian forest edges in Southern Amazonia

**DOI:** 10.1038/s41598-023-39510-x

**Published:** 2023-08-01

**Authors:** Leonardo Maracahipes-Santos, Divino Vicente Silvério, Leandro Maracahipes, Marcia Nunes Macedo, Eddie Lenza, Kathi Jo Jankowski, Michelle Y. Wong, Antônio Carlos Silveiro da Silva, Christopher Neill, Giselda Durigan, Paulo Monteiro Brando

**Affiliations:** 1 Instituto de Pesquisa Ambiental da Amazônia (IPAM), Rua Horizontina 104, Centro, Canarana, MT 78640-000 Brazil; 2 Programa de Pós-Graduação em Ecologia e Conservação, Universidade do Estado de Mato Grosso (UNEMAT), Campus de Nova Xavantina, Rua Prof. Dr. Renato Figueiro Varella, Caixa Postal 08, Nova Xavantina, MT 78690-000 Brazil; 3 Departamento de Biologia, Universidade Federal Rural da Amazônia (UFRA), Capitão Poço, Pará 68650-000 Brazil; 4 Department of Plant Biology, Institute of Biology, University of Campinas (UNICAMP), P.O. Box 6109, Campinas, SP 13083‐970 Brazil; 5 Woodwell Climate Research Center, Falmouth, MA 02450 USA; 6 U.S. Geological Survey Upper Midwest Environmental Sciences Center, La Crosse, WI 54603 USA; 7 Cary Institute of Ecosystem Studies, Millbrook, NY USA; 8 Laboratório de Ecologia e Hidrologia, Instituto de Pesquisas Ambientais, Floresta Estadual de Assis, Assis, SP Brazil; 9 Yale School of the Environment, Yale University, New Haven, CT USA

**Keywords:** Ecosystem ecology, Forest ecology

## Abstract

Tropical forest fragmentation from agricultural expansion alters the microclimatic conditions of the remaining forests, with effects on vegetation structure and function. However, little is known about how the functional trait variability within and among tree species in fragmented landscapes influence and facilitate species’ persistence in these new environmental conditions. Here, we assessed potential changes in tree species’ functional traits in riparian forests within six riparian forests in cropland catchments (Cropland) and four riparian forests in forested catchments (Forest) in southern Amazonia. We sampled 12 common functional traits of 123 species across all sites: 64 common to both croplands and forests, 33 restricted to croplands, and 26 restricted to forests. We found that forest-restricted species had leaves that were thinner, larger, and with higher phosphorus (P) content, compared to cropland-restricted ones. Tree species common to both environments showed higher intraspecific variability in functional traits, with leaf thickness and leaf P concentration varying the most. Species turnover contributed more to differences between forest and cropland environments only for the stem-specific density trait. We conclude that the intraspecific variability of functional traits (leaf thickness, leaf P, and specific leaf area) facilitates species persistence in riparian forests occurring within catchments cleared for agricultural expansion in Amazonia.

## Introduction

Ongoing deforestation, fragmentation, and climate change are imminent threats to tropical forests^1,2^. Clearing of tropical forests for agriculture and cattle ranching promotes local^3,4^ and regional^5–7^ changes in environmental conditions^8,9^. These changes have pervasive impacts on remaining forests, including edge effects^10^, changes in solar radiation^9^, and increases in surface temperature^6^. Such changes affect forest composition, species richness^10,11^, and functional diversity, with potential impacts on ecosystem services such as carbon storage^12–14^ and evapotranspiration^6^.

**Figure 1:**
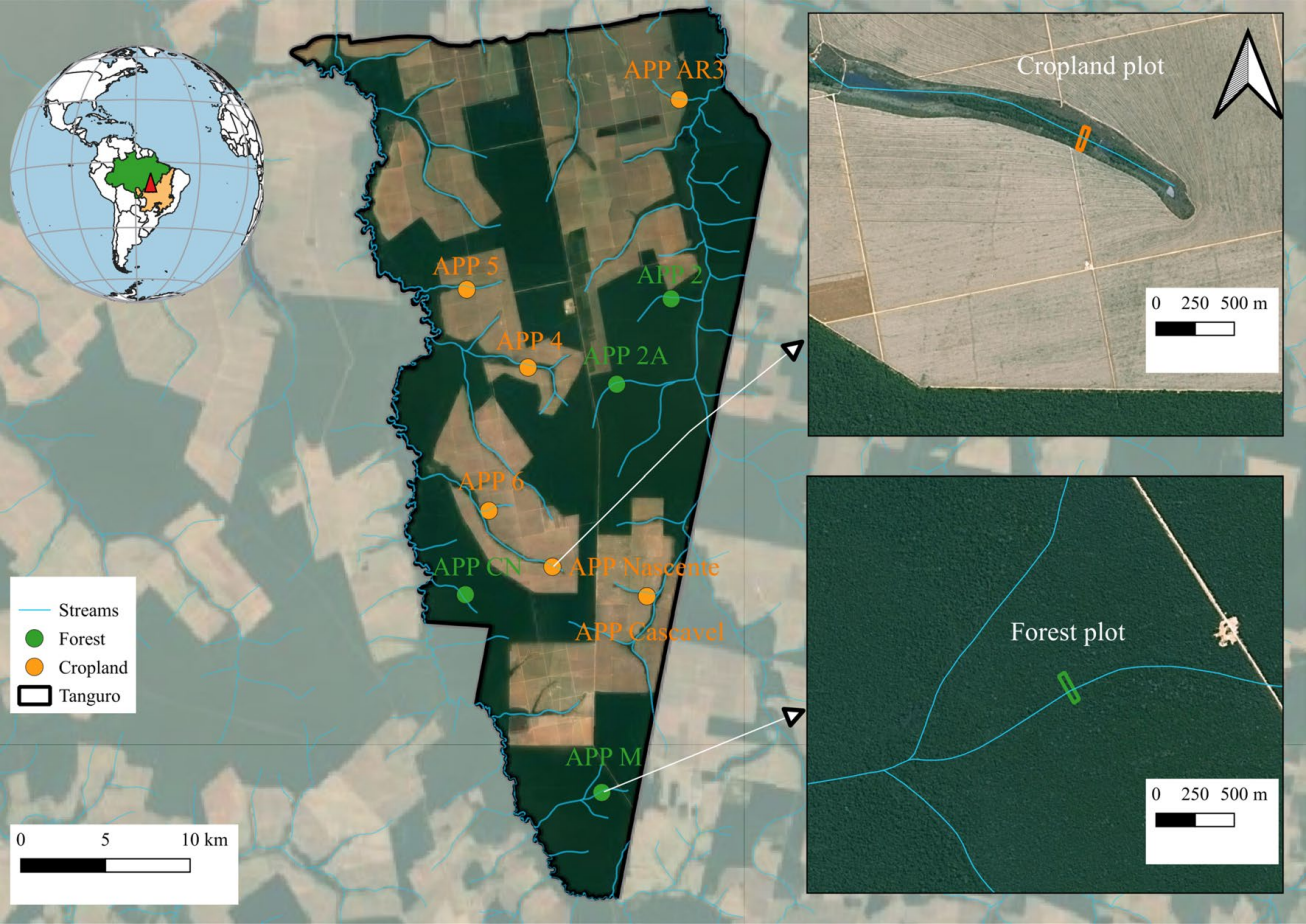
Location of tree communities in riparian forests in forested catchments (Forest = green circle) and riparian forests in cropland catchments (Cropland = orange circle) in the southern Amazonia, Querência–MT, Brazil. Left: Fazenda Tanguro; Right: example of one a riparian forest transect in cropland catchments transect (top) and a riparian forest transect in forested catchments (bottom). We create the map and the globe in QGIS version 3.30 (https://www.qgis.org/pt_BR/site/forusers/download.html).

Despite the importance of native forests for climate regulation, deforestation in Amazonia has intensified in recent decades, primarily for the expansion of agriculture and cattle ranching in the south and east—a region known as the “arc of deforestation”^15–19^. Riparian forest fragments persisting in these agricultural landscapes have played a disproportionate role in preserving terrestrial habitat corridors and the integrity of streams and rivers. Conversely, the degradation of riparian forests may impair stream ecosystems, with negative consequences for local communities^17,20–22^. Maintaining the critical ecological and ecosystem services provided by riparian forests in cropland catchments^10,11,19^ requires a nuanced understanding of the cause-and-effect relationships between tropical forest conversion and fragmentation, as well as the functional processes of vegetation in these fragmented landscapes. Remnants of riparian forest associated with small streams in Amazonia afford us an opportunity to study potential changes in the functional traits of these species over time (Fig. 1).

The rapid pace of environmental change associated with deforestation in southern Amazonia could preclude species adaptation and cause changes in forest function^14^. Anthropogenic disturbances associated with deforestation and fragmentation drive changes in species composition and often select for species with a greater capacity to adapt to novel environmental conditions^23,24^. In general, species with higher intraspecific variability in their traits are more likely to persist in modified environments, while others may become locally extinct^23,24^. These local extinctions can compromise critical services provided by tropical forests. Additionally, fragmentation facilitates the colonization of pioneer/generalist species^4,27^ in these human-disturbed environments, which can drive changes in mechanisms related to resource acquisition and competition among tree species^4,24,25^. Previous research indicates that these processes can act synergistically, leading to the assembly of new riparian tree communities, with associated changes in species diversity, composition, and vegetation structure^10^. Evidence from subtropical forests in Mexico suggests that environmental filtering and functional traits drive such changes in community assembly^25^, but these mechanisms have yet to be examined in riparian forests of the tropical Amazon.

Considering that different tree species may exhibit contrasting ecological strategies in response to fragmentation, it is essential to evaluate species-specific functional responses to fragmentation and edge effects. Quantifying the functional responses of plant communities to fragmentation permits a better understanding of how ecosystem-level processes may shift in response to environmental change. For example, some species adopt more conservative strategies to maximize their resource economy in old-growth forests, whereas others tend to adopt acquisitive strategies to optimize resource use following disturbance^26,27^. Acquisitive strategies in woody trees are related to competition for available resources and imply a high investment in tree height, specific leaf area, and low wood density^8,9,28–30^. A better understanding of how species adapt to fragmented cropland catchments using different life strategies will be crucial for predicting the future trajectories of tropical forests under increasing degradation pressure.

Although conservative strategies typically characterize primary tropical forests, increased anthropogenic disturbances may cause tree communities to adopt more acquisitive strategies^9^ owing to changes in light and resource availability (e.g., water and nutrients)^31,32^. These alterations in functional strategies can be caused by the increased dominance of pioneer species^33^, which have fast growth, high mortality and recruitment rates, and a short lifespan. For example, in riparian forests in the transition zone between the Cerrado and Amazonia, *Tachigali vulgaris* is a colonizing generalist that recruits at a high rate and is abundant in forest gaps or burned forests^33,34^. Changes in functional strategies can also result from the increased dominance of generalist species, which can have high intraspecific variability that allows them to thrive in a wide range of environmental conditions. Both species turnover and intraspecific variability can play important roles in driving changes in functional diversity and the associated ecosystem functions and services.

In this study, we aimed to assess whether a broader range of functional trait values facilitates species persistence in fragmented riparian forests (i.e., those found along streams in cropland catchments) in southern Amazonia. Considering that deforestation promotes changes in the structure and species diversity of cropland riparian forests^10,11^, we evaluated whether the functional traits of riparian forest communities also changed in response to land-use changes and fragmentation. More specifically, we tested four hypotheses: *H1:* Functional traits of riparian forest communities differ between riparian forests in cropland catchments (Cropland) and riparian forests in forested catchments (Forest) due to the intraspecific variability of species shared between the two environments (plasticity), but also due to colonization of riparian forests in cropland catchments by pioneer species (species turnover); *H2:* Tree species occurring in both riparian forests in cropland catchments and riparian forests in forested catchments exhibit higher intraspecific variability of leaf and stem functional traits, allowing those species to persist in or colonize fragmented landscapes; *H3:* Community functional traits are more similar between riparian forests in cropland catchments (Cropland) and riparian forests in forested catchments (Forest) close to the stream, where environmental filters associated with edge effects near agricultural fields are less intense; *H4*: Tree assemblages growing near agricultural fields tend to be dominated by species with acquisitive strategies, given that resources (e.g., light, nutrients) may be more abundant, while filters associated with agricultural fields (e.g., increases in surface temperature, winds) may select species with more conservative strategies for survival.

## Material and methods

### Study area

This study was conducted in riparian forests at Fazenda Tanguro, located in the municipality of Querência, Mato Grosso (MT) state, Brazil. For a detailed description of the study area, tree community, and species diversity, refer to Maracahipes-Santos et al.^8^. Briefly, we sampled the functional traits of riparian forest tree species in four different forested catchments with no evidence of recent disturbance (“Forest”) distributed across the landscape in the study area (Fig. 1). We also sampled the functional traits of riparian forest tree species in six different catchments covered mostly by croplands (“Cropland”; Fig. 1). The number of catchments sampled were based on the availability of forested catchments in the farm. All cropland catchments share a similar history of disturbance, as described below. Although cropland riparian forests have never been cleared, they have experienced edge effects for more than three decades. The climate in this region has two well-defined seasons (Fig. S1): a rainy season from October to April and a dry season from May to September, with annual precipitation ranging from 1900 to 2200 mm, and average temperature from 24 to 26 °C^35^ (Fig. S1).

At Fazenda Tanguro, the structure of riparian forests today bears the legacy of 42 years of land-use and land-cover change. Like other areas of the Amazon-Cerrado agricultural frontier, deforestation at our field site began during the 1980s for the expansion of cattle ranching^36^. Typically, cattle use riparian zones to access drinking water in streams, which is likely to compact soils, trample regenerating vegetation, and alter stream water quality^5^. In the early 2000s, large-scale agricultural production replaced pastures with soybean monoculture, followed by double-cropping of soybeans and corn, and as recently as 2019 by cotton cultivation. At Fazenda Tanguro, the sampled riparian forests in deforested catchments experienced cattle and edge effects for more than three decades, until the replacement of pasture by cropland in 2007—a land use trajectory that is quite typical of the broader region. Given the broader forests protecting riparian zones in forested catchments and the absence of any signal of other disturbances, we assumed that cattle had no impact on intact riparian forests.

### Data collection

We characterized the functional traits of riparian forest communities at the peak of rainy season (November 2017 to January 2018). Following Pérez-Harguindeguy et al.^37^, we sampled the following functional traits: maximum tree height (Hmax), stem-specific density (SSD), relative bark thickness (weighted by trunk diameter at breast height) (BT), leaf thickness (LT), leaf area (LA), specific leaf area (SLA), and leaf concentrations of phosphorus (P), potassium (K), nitrogen (N), carbon (C), calcium (Ca), and magnesium (Mg) (Table S1). For each riparian forest type (forest and cropland), we sampled ten randomly selected individuals of each species within the plots (Table S2). For locally rare species, we sampled additional randomly selected individuals outside the plot (Table S2) to guarantee a minimum sample size of three individuals. With this adaptation, we sampled the minimum variability in the mean of these species. To determine leaf nutrient concentrations, we sampled three individuals from each species per environment (Table S1). The functional traits selected here are associated with strategies for rapid acquisition or conservation of resources^29^, as well as tree responses to disturbances such as water and nutrient stress^37^. Leaf nutrient concentrations were measured at the Laboratory of Plant Analysis (Forestry Engineering Department—UFV, Viçosa, MG, Brazil).

### Data analysis

We compared the functional traits of species occurring in two riparian forest types (i.e., those in forested or cropland catchments) with a high degree of dissimilarity^10^, considering the whole community (all species from each environment), species unique to each forest type, and species shared between the two forest types as described below.

To compare community functional traits between croplands and forests, we used a multivariate analysis of variance (MANOVA), applying the *Manova* function from the base package *“stats”* in R^38^. We also used MANOVA to compare the mean traits of unique species and species shared by both forest types. To quantify the effect of land use and distance from the stream (within the fragment) on the observed functional traits, we used generalized mixed models (GLMM; *lmer* function from the “*lme4*” package^39^). We checked for multicollinearity among the functional traits of riparian forests (n = 12) using variance inflation factors (VIFs) and the *vifstep* function of the “*usdm*” package^40^. We found no evidence of collinearity (VIF ≤ 3 for all variables^41^), so we retained all functional traits in our analysis (Table S3). We applied a Principal Component Analysis (PCA) to evaluate the interrelationships in our set of functional traits for the two forest types separately, using the *princomp* function from the base package “*stats*” in R^38^. In this analysis, we used the *decostand* function with the standardized method from the “*vegan*” package^42^ to standardize the variables. We further evaluated the differences between the two forest types based on the first two axes of the PCA using a linear model implemented in the *lm* function from the base package “*stats*” in R^38^.

Finally, we used the Sum of Squares (SS) decomposition approach by Lepš et al.^43^ to disentangle the relative importance of species turnover and intraspecific variability in driving potential differences in functional traits between forest and cropland riparian forests. To do so, we calculated: (1) the community-weighted ‘specific’ average for each trait (using trait values of each species within each riparian forest), which is the variation that can be caused by both species turnover and intraspecific trait variability; (2) the community weighted ‘fixed ’ average for each trait, using mean trait values of each species for the riparian forest where it does occur; and (3) the ‘intraspecific variability’ as the difference between ‘specific’ and ‘fixed ’ average traits. Next, we used a two-way ANOVA to attribute the explained variation in trait values to species turnover, intraspecific variability, and their interaction. We used the *decompCTRE* function in the *cati* R package for this analysis^44^.

Finally, given the presence of some outliers in our dataset, we conducted bootstrapping analyses (at 95% confidence interval) to test the reliability of our conclusions. Briefly, we removed the outliers and resampled data to compare traits in the entire tree communities for Cropland versus Forest riparian forests, as well as species common to both environments and species unique to each environment (see Supplementary Material for details).

### Additional statements

The sample collection used to evaluate leaf nutrient concentrations in this study followed all relevant institutional and national legislation and guidelines. Our license to collect sample in the field is registered in the Sistema de Autorização e Informação em Biodiversidade—SISBIO (https://www.icmbio.gov.br/cpb/index.php/sisbio) under Number 70661-1.

## Results

**Figure 2:**
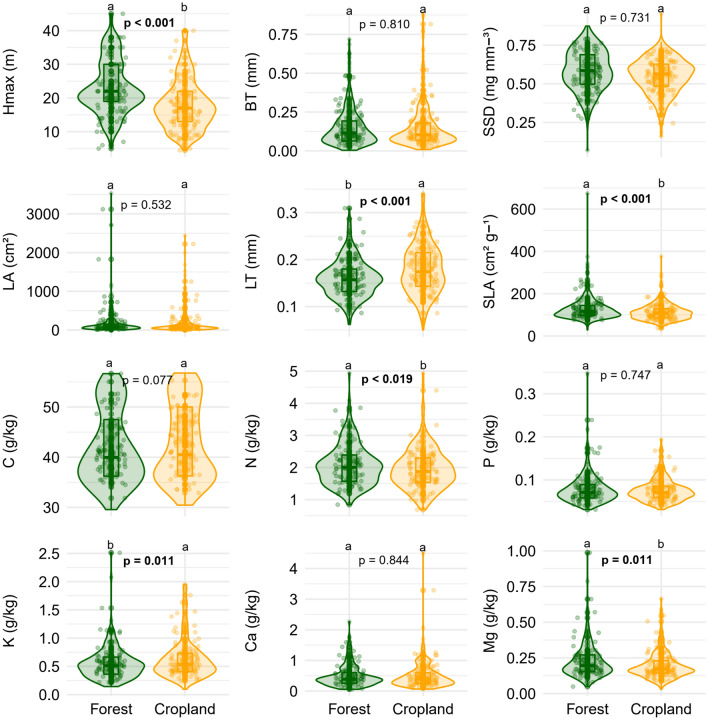
Mean functional traits of riparian forest tree communities in forested catchments (Forest) and cropland catchments (Cropland) in southern Amazonia, Querência–MT, Brazil. Hmax = maximum tree height; BT = bark thickness; SSD = stem-specific density; LA = leaf area; LT = leaf thickness, SLA = specific leaf area, C = leaf carbon concentration, N = leaf nitrogen concentration, K = leaf potassium concentration, P = leaf phosphorus concentration, Ca = leaf calcium concentration, and Mg = leaf magnesium concentration. Letters indicate significant differences by Multivariate Analysis of Variance (MANOVA) at a 5% significance level.

**Figure 3:**
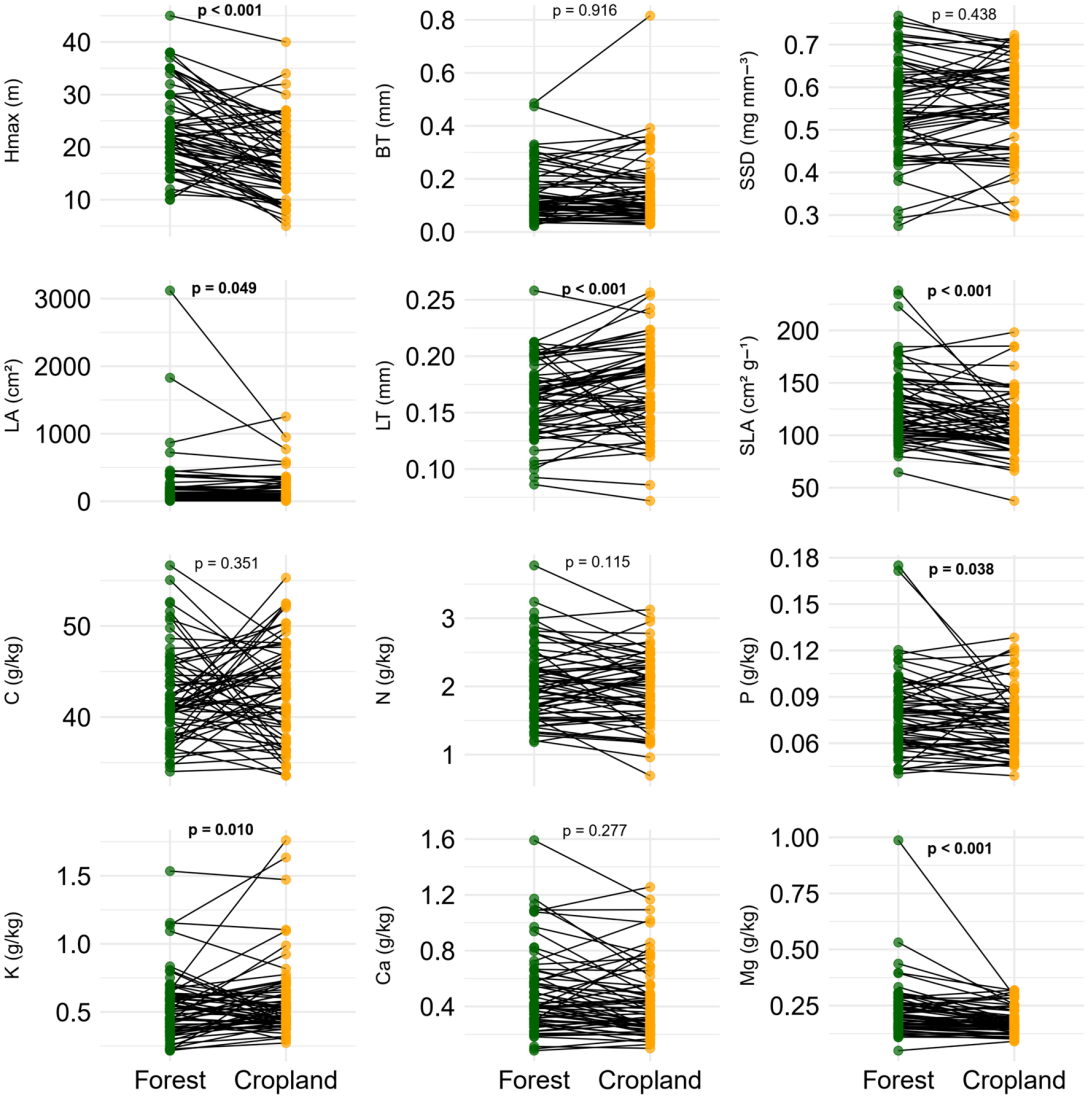
Comparisons of mean values of functional traits of riparian tree species shared between riparian forests in forested (Forest) and cropland (Cropland) catchments in southern Amazonia, Querência–MT, Brazil. Points indicate individual species, with lines connecting trait values for a species found in cropland vs. forested catchments. Hmax = maximum tree height; BT = bark thickness; SSD = stem-specific density; LA = leaf area; LT = leaf thickness, SLA = specific leaf area, C = leaf carbon concentration, N = leaf nitrogen concentration, K = leaf potassium concentration, P = leaf phosphorus concentration, Ca = leaf calcium concentration, and Mg = leaf magnesium concentration. A 5% significance level was used for this analysis (n = 64).

**Figure 4:**
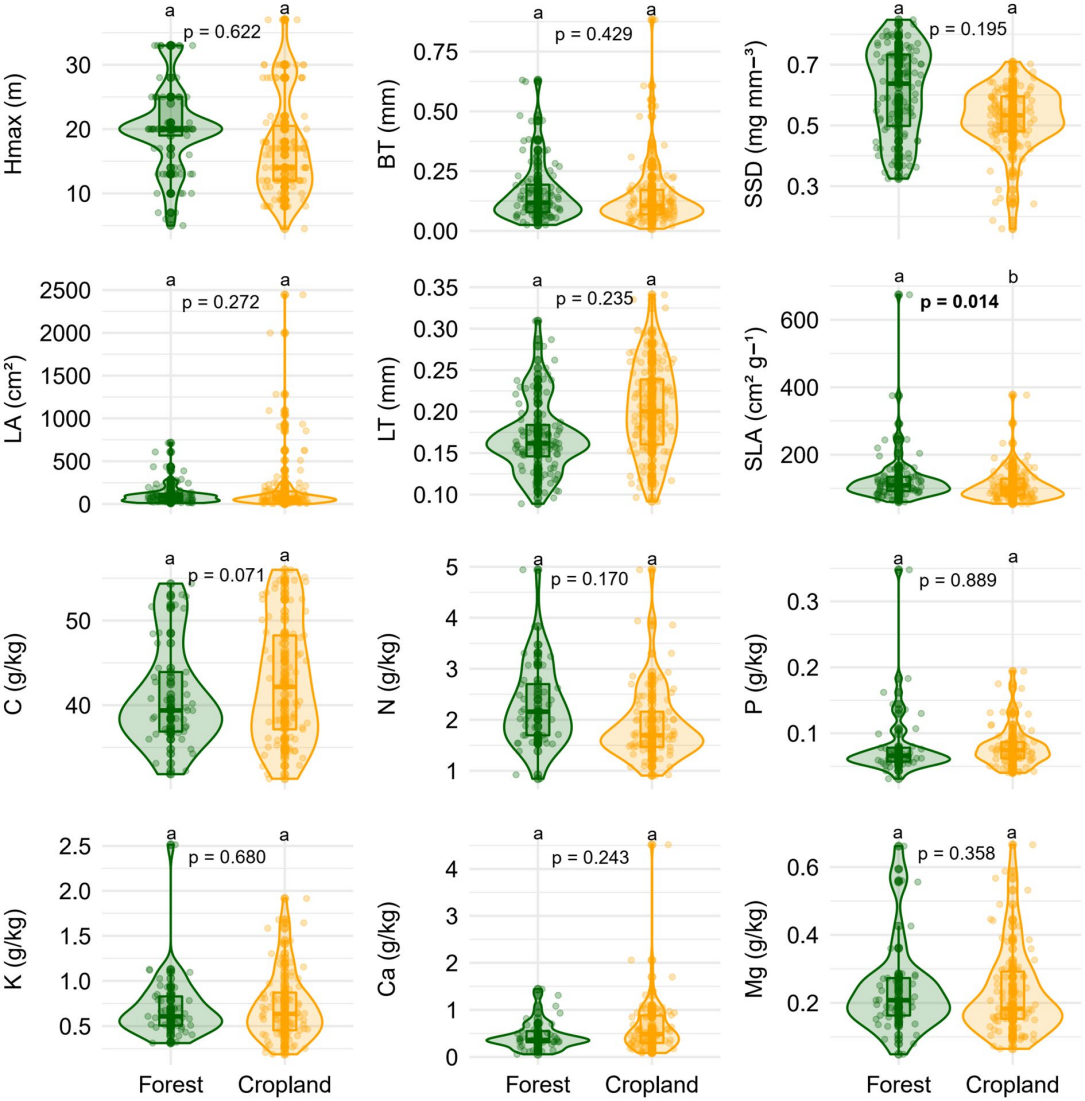
Mean values of functional traits of species restricted (unique) to riparian forests in forested (Forest) or cropland (Cropland) catchments in southern Amazonia, Querência–MT, Brazil. Hmax = maximum tree height; BT = bark thickness; SSD = stem-specific density; LA = leaf area; LT = leaf thickness, SLA = specific leaf area, C = leaf carbon concentration, N = leaf nitrogen concentration, K = leaf potassium concentration, P = leaf phosphorus concentration, Ca = leaf calcium concentration, and Mg = leaf magnesium concentration. Letters indicate significant differences by Multivariate Analysis of Variance (MANOVA) at a 5% significance level.

**Figure 5:**
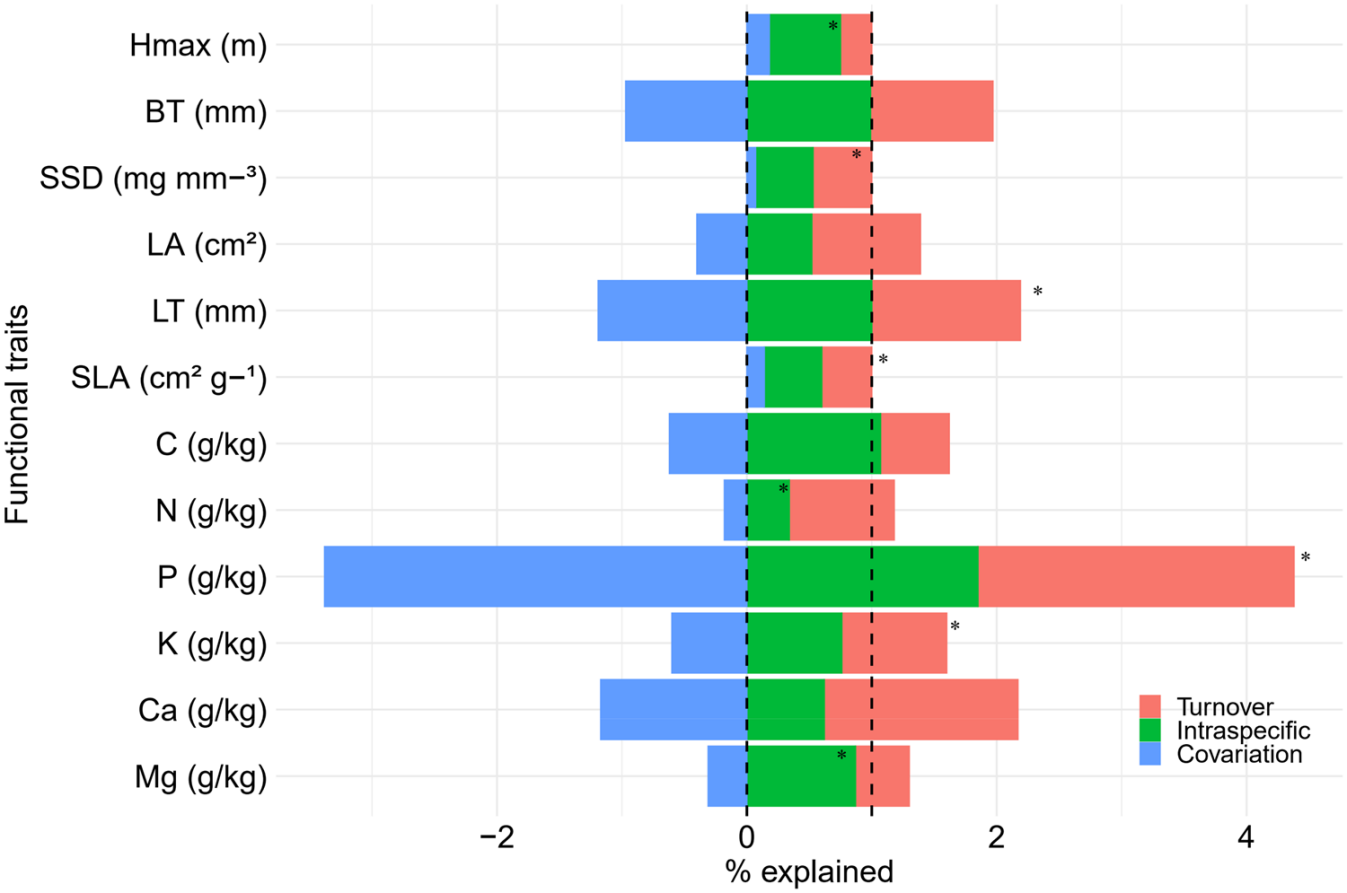
Effect of turnover on tree species composition, intraspecific trait variability, and their covariation between riparian forests in forested (Forest) and cropland (Cropland) catchments in southern Amazonia, Querência–MT, Brazil. Hmax = maximum tree height; BT = bark thickness; SSD = stem-specific density; LA = leaf area; LT = leaf thickness, SLA = specific leaf area, C = leaf carbon concentration, N = leaf nitrogen concentration, K = leaf potassium concentration, P = leaf phosphorus concentration, Ca = leaf calcium concentration, and Mg = leaf magnesium concentration. Line dashed = Total—specific average [0 to 1]; * = Differed significantly at 5% (Table S5).

Overall, we found that six of the twelve functional traits measured in our study significantly differed between riparian forests in forested catchments (forest) and riparian forests in cropland catchments (cropland) when we considered all sampled species (Fig. 2): maximum tree height [Hmax], leaf thickness [LT], specific leaf area [SLA], N, K, and Mg. Differences in functional traits between intact forest and cropland were associated with intraspecific variability (Hmax, N, Mg), species turnover (SSD), and the synergy between the two processes (LT, SLA, P, K; Fig. 5, Table S8). Although species turnover played an important role in explaining trait variability, we found differences between forest types for eight traits (Hmax, LA, LT, SLA, P, K, Ca, and Mg) when comparing only species common to both environments (Fig. 3). On the other hand, for the exclusive species, only SLA was significantly different between the two environments (Fig. 4). These overall results, presented in detail below, suggest strong fragmentation effects on the functional traits of tree communities and a higher contribution of intraspecific variability to differences observed between the two studied environments.

Our Principal Component Analysis (PCA) showed important differences in tree communities between riparian forests in forested catchments and riparian forests in cropland catchments (Fig. S2; From a MANOVA: F_(1,184)_ = 4.36; *p* = 0.038). With the first two axes explaining 40.3% of the variation in functional traits, our PCA showed that the differentiation between forests and croplands was associated with high SLA, N, and P of forests, as well as with high stem-specific density (SSD), and leaf thickness (LT) in croplands.

Consistent with our first hypothesis (*H1*), riparian forests in forested catchments had higher values for four out of the six functional traits that differed between sites: (a) maximum tree height (21.8% higher in forested than riparian forests in cropland catchments); (b) specific leaf area (11.7%); (c) leaf magnesium concentration (13.5%); and (d) leaf nitrogen concentration (5.3%) (Fig. 2, Table S4). In contrast, two traits showed higher values in cropland than in riparian forests in forested catchments: leaf thickness (11.0%) and leaf potassium concentration (13.1%) (Fig. 2, Table S4). Relative bark thickness, stem-specific density, leaf area, carbon, phosphorus, and calcium concentrations did not differ between forest types at the community level (Fig. 2, Table S4).

We found that the observed differences in functional traits at the community level (all species) were mainly driven by differences in the functional traits of the 64 species shared by the two forest types. Two comparisons between the results of different analyses reinforce the following: First, five of the six traits with significant differences between the studied environments at the community level (maximum tree height (Hmax), leaf thickness (LT), specific leaf area (SLA), K, and Mg) (Fig. 2, Table S4) also differed between forest and cropland when just the shared species were considered in the analysis (Fig. 3, Table S6); Second, our decomposition of variance analysis showed that in four of the six traits (Hmax, N, K, and Mg) the intraspecific variability was more important for observed differences between environments, whereas in one trait (LT) it was due to the interaction between intraspecific variability and species turnover (Fig. 5, Table S5).

To test whether differences between the two types of forest are determined by intra-specific variability, turnover, or both, we conducted a sum of squares decomposition analysis (Fig. 5, Table S5). We found significant differences between forest and cropland for seven of twelve traits analyzed. These differences were explained mainly by intraspecific variability (Hmax, N, and Mg) or by synergy between intraspecific variability and turnover (LT, SLA, P, and K). In contrast, turnover explained only the differences for SSD (Fig. 5, Table S5). Thus, differences in plant functional adjustments between the two forest types were best explained by variations in attributes (n = 3) among those species common to both forests and croplands. However, the synergistic effect between intraspecific variation and species turnover on functional attributes (n = 4) also played an important role in explaining differences between catchments for some traits.

Consistent with our first hypothesis (*H1*), riparian forests in forested catchments had higher values than cropland catchments for five out of the seven functional traits differing between sites: maximum tree height, leaf area, specific leaf area, leaf phosphorus concentration, and leaf magnesium concentration (Fig. 3, Table S6). In contrast, two traits showed higher values in riparian forests in cropland catchments than in forested catchments: leaf thickness and leaf potassium concentration (Fig. 3, Table S6). Contrary to our second hypothesis (*H2*), we found similar functional trait values (11 of 12 studied) when comparing species unique to either riparian forests in forested catchments (26) or riparian forests in cropland catchments (33). The only trait that differed significantly between the two forest types was specific leaf area (SLA), with higher values observed in forested catchments (128.9 cm^2^ g^−1^) compared to cropland catchments (average = 109.3 cm^2^ g^−1^; Fig. 4, Table S7; F_(1,48)_ = 6.46; *p* = 0.014). In summary, individuals in riparian forests in forested catchments were taller, produced thinner leaves, had higher P and Mg concentrations, and had lower K concentrations (Figs. 3, S3, Table S6), and these differences were due mainly to intraspecific variability rather than species turnover.

Our third hypothesis (*H3*) received strong support, given that our GLMM statistical model showed three functional traits changing as a function of distance from the stream channel (bark thickness [BT], stem-specific density [SSD], and phosphorus [P]) and these differed between forest types. However, the differences between forest types were small relative to the variability of the data as a function of species (Fig. S4, Table S8). We also found that both forest types showed increases in specific leaf area (SLA), N, P, and Mg values, but decreases in maximum tree height (Hmax), leaf thickness (LT), and Ca with increasing distance from the stream (Fig. S4, Table S8).

Finally, when we considered all the comparative analyses of the 12 attributes between riparian forests in forested and cropland catchments (except for comparisons between species unique to each forest type), we found that five functional traits (Hmax, LT, SLA, K, and Mg) always differed between the two forest types (Figs. 2, 3, 5, S3–S4). In general, tree species in cropland catchments were smaller and had thicker leaves, with higher specific leaf area and leaf potassium content, and with lower nitrogen and leaf magnesium content, compared to riparian forest trees in forested catchments. These results support our fourth hypothesis (*H4*), since larger plants with thinner leaves, higher SLA and leaf magnesium content represent strategies to increase light acquisition efficiency. Also, the higher leaf potassium content in riparian forests in cropland catchments can be interpreted as an efficient strategy to deal with water stress (Table S1).

Our reliability analysis (bootstrapping at 95% CI) confirmed most of the findings reported above. For instance, in the comparison of the entire community, the SSD trait was found to be significantly different between riparian forests in Forest and Cropland catchments (Fig. S5). When considering only the shared species, SSD was also found to differ between environments (Fig. S5). For species unique to each environment, the traits Hmax, SSD, LA, LT, C, N, and Ca exhibited significant differences between environments (Fig. S5). In two cases, the bootstrapping analysis was inconsistent with differences observed in the analyses presented above: P, and LA for shared species to a given environment (Fig. S5).

## Discussion

Previous studies have shown that fragmentation in Amazonia reduces species richness in riparian forests, alters species composition^10,11^, and decreases biomass stock^45^. Here, we show that agricultural practices also drive fundamental changes in the functional traits of tree species in riparian forests. Specifically, we found that changes in functional traits in the agricultural frontier of Amazonia were primarily associated with higher intraspecific variability of species occurring in both forested and cropland catchments than in species unique to either forest type. This high intraspecific variability may explain the persistence of some tree species in riparian forests in highly disturbed environments. Our results also support the hypothesis that the functional traits of trees located close to stream channels in cropland riparian forests, where edge effects are expected to be less intense, are more similar to those of riparian forests in forested catchments.

An important finding was that riparian tree species common to both forested and cropland catchments showed high intraspecific variability. For seven of the twelve functional traits studied, generalist species had higher intraspecific variation than environment-specific specialists. Conversely, tree functional traits were surprisingly similar for tree species restricted to either environment, with the exception of SLA (Fig. 4). Tree species unique to either environment were locally rare (n < 3 individuals), which raises the possibility of major decreases in population size for habitat-specialists occurring in riparian forests in cropland catchments, with the potential of decreased richness. We speculate that there is an ongoing functional homogenization in croplands due to losses of such species, as indicated by the six traits that differed between the two forests in the community-level analyses. This underscores the importance of evaluating the specific ecosystem services represented by these rare species, although that is beyond the scope of this present study.

While the high intraspecific variability among generalist species may allow some of them to persist in (or colonize) riparian forests in cropland catchments, it may also have negative impacts on ecosystem function. For example, some fast-growing pioneer species with lower wood density may decrease total carbon stocks in cropland forests^10^ and hamper regulation of local and regional temperatures^6,13,14,19^. One evidence of fast-growing species entrance in our study was that wood density was the only variable associated with pure species turnover effects in the sum-of-squares analysis, indicating a replacement of slow-growing species (late successional stage) by fast-growing species (early successional stage), which may result in taxonomic and functional homogenization of riparian forests in cropland catchments.

At the community level, tree species in riparian forests in forested catchments were taller and produced leaves with higher specific leaf area, leaf magnesium and nitrogen concentrations than riparian forests in cropland catchments. The mechanisms accounting for the presence of taller trees in riparian forests in forested catchments are uncertain but probably relate to higher competition for light in dense forests and lower windstorm severity in riparian forests in forested catchments than in riparian forests in cropland catchments. At the same time, differences in SLA between environments are probably linked to environmental filters in agricultural catchments, selecting for trait values of some species that permit higher assimilation of light and water availability in cropland^17^. Trees exposed to higher solar radiation develop a thicker mesophyll and smaller leaves, which would explain the lower SLA values in riparian forests in cropland catchments^8,9,46^. However, it is possible that the legacy of cattle in utilizing riparian areas to access drinking water from streams may have influenced species composition and functional diversity by compacting soils and trampling regenerating vegetation^5^.

Among all the traits investigated here, leaf traits such as nutrient content (N, P, K, and Mg), leaf thickness, and specific leaf area are the clearest indicators of forest responses to land-use change, particularly compared to whole-tree traits such as height and stem-specific density (SSD). Leaves are organs that demonstrate high plasticity in their traits and therefore can be responsive to subtle environmental changes on a shorter time scale. Although we recommend using leaf traits to evaluate the consequences of fragmentation for tree ecological adjustments, our comparisons of traits among species unique to each environment did not detect meaningful differences between them. Community- and shared-species analyses may be more effective than species-level analyses for assessing tree adjustments to new conditions imposed by habitat fragmentation.

Our results show that deforestation and edge effects caused changes in the ecological strategies of tree species in riparian forest fragments adjacent to agricultural areas in southern Amazonia. Under these conditions, conservative strategies may indicate some level of adaptation (by resistant or generalist species) to environmental stress and/or responses to degraded environments. In contrast, acquisitive strategies emerge from newly recruited species, which tend to be fast-growing pioneers. Our results show that the functional traits that best express the effects of changing environmental conditions were maximum tree height, leaf thickness, specific leaf area, and leaf nutrient concentrations (nitrogen, potassium, and magnesium). On the other hand, we showed that species functional traits, such as greater leaf thickness and higher P content, suggest resource conservation strategies in riparian forests in cropland catchments compared to riparian forests in forested catchments.

We suggest that the observed changes in species richness and composition^10,11^ and functional traits of riparian forest tree species (this study) alter ecosystem functions and may compromise the maintenance of crucial ecosystem services, including but not limited to the reduction of carbon stocks in communities adjacent to agricultural areas^12–14,34,47^. In this context, long-term monitoring of the functional responses of these communities to the new conditions imposed by deforested edges is necessary.

We conclude that the study of functional traits, widely adopted in the last two decades, is an essential tool for evaluating the effects of deforestation on tree communities and provides a critical perspective for understanding tree phenotypic intraspecific variability and responses to environmental filters^8,9^. Our results provide new insights into how land use change reduces the quality of crucial ecosystem services provided by riparian forests, such as carbon storage^12–14^ and evapotranspiration^6^. Given the great ecological importance of riparian forests, increased efforts are needed to study the functional responses of species to forest degradation, because changes can occur rapidly without sufficient time for many species to adapt. Further research on the functional traits of species and communities could provide a helpful perspective on the constraints for species establishment and survival in these degraded landscapes—and facilitate more strategic restoration strategies that target specific ecosystem functions and are more resilient to environmental filters.

## Supplementary Information

Supplementary Information.

## Data Availability

The datasets generated during and/or analysed during the current study are available from the corresponding author on reasonable request.
