# Supplementary material for: Intraspecific trait variability facilitates tree species persistence along riparian forest edges in Southern Amazonia
Source: Sci Rep. 2023 Aug 1;13:12454. doi: 10.1038/s41598-023-39510-x (PMC10394016; doi:10.1038/s41598-023-39510-x)
Supplement: Supplementary file 1 — Supplementary Information. [file 41598_2023_39510_MOESM1_ESM.docx]

Electronic Supplemeantal Material

**Intraspecific trait variability facilitates tree species persistence along riparian forest edges in Southern Amazonia**

Leonardo Maracahipes-Santos ^1,2,10^, Divino Vicente Silvério ^1,2,3^, Leandro Maracahipes ^1,4^, Marcia Nunes Macedo ^1,5^, Eddie Lenza ^2^, Kathi Jo Jankowski ^6^, Michelle Y. Wong ^7^, Antônio Carlos Silveiro da Silva ^1^, Christopher Neill ^5^, Giselda Durigan ^8^, and Paulo Brando ^1,2,5,9^

**Author Contributions:** L.M.-S., D.V.S., L.M., M.N.M., E.L., K.J.J., M.Y.W., A.C.S.S., C.N., G.D., and P.B. conceived and designed the experiments. L.M.-S., D.V.S., L.M., M.N.M., E.L., and P.B. performed the experiments. L.M.-S., D.V.S., L.M., M.N.M., C.N., G.D., and P.B. analyzed the data. L.M.-S., D.V.S., L.M., M.N.M., E.L., K.J.J., M.Y.W., A.C.S.S., C.N., G.D., and P.B. wrote the manuscript; other authors provided editorial advice.

^1^ *Instituto de Pesquisa Ambiental da Amazônia (IPAM), Rua Horizontina 104, 78640-000, Canarana, MT, Brazil*

^2^ *Programa de Pós-graduação em Ecologia e Conservação, Universidade do Estado de Mato Grosso (UNEMAT), Campus de Nova Xavantina, Rua Prof. Dr. Renato Figueiro Varella, Caixa Postal 08, 78690-000, Nova Xavantina, MT, Brazil*

*^3^ Departamento de Biologia, Universidade Federal Rural da Amazônia (UFRA), Capitão Poço 68650-000, Pará, Brazil*

^4^ *Department of Plant Biology, Institute of Biology, P.O. Box 6109, University of Campinas (UNICAMP), Campinas, 13083‐970, SP, Brazil*

^5^ *Woodwell Climate Research Center, Falmouth, MA 02450, USA*

^6^ *U.S. Geological Survey Upper Midwest Environmental Sciences Center, La Crosse, WI 54603, USA*

*^7^ Cary Institute of Ecosystem Studies, Millbrook, NY, USA*

*^8^ Laboratório de Ecologia e Hidrologia, Instituto de Pesquisas Ambientais, Floresta Estadual de Assis, Assis, SP, Brazil*

*^9^ Yale School of the Environment, Yale University, New Haven, CT, United States.*

*^10^ Corresponding author at: Instituto de Pesquisa Ambiental da Amazônia (IPAM), 78640-000, Rua Horizontina, 104, Centro, Canarana, MT, Brazil. E-mail address: maracahipesbio@gmail.com (L. Maracahipes-Santos).*

Any use of trade, firm, or product names is for descriptive purposes only and does not imply endorsement by the U.S. Government.

**ORCIDS:**

Leonardo Maracahipes-Santos (Orcid ID: 0000-0002-8402-1399)

Divino Vicente Silvério (Orcid ID: 0000-0003-1642-9496)

Leandro Maracahipes (Orcid ID: 0000-0002-6148-3291)

Marcia Nunes Macedo (Orcid ID: 0000-0001-8102-5901)

Eddie Lenza (Orcid ID: 0000-0001-9139-5949)

Kathi Jo Jankowski (Orcid ID: 0000-0002-3292-4182)

Michelle Y. Wong (Orcid ID: 0000-0002-7830-8035)

Antônio Carlos Silveiro da Silva (Orcid ID: 0000-0002-8180-825X)

Christopher Neill (Orcid ID: 0000-0002-0232-8371)

Giselda Durigan (Orcid ID: 0000-0003-0693-3154)

Paulo Monteiro Brando (Orcid ID: 0000-0001-8952-7025)

**Additional information:**

**Data Collection**

We collected functional traits at the height of the rainy season (November 2017 to January 2018), when we expected maximum leaf expansion, maturity, and peak structural tree growth^1^. Following the protocol of Pérez-Harguindeguy et al.^1^, we sample the following functional traits:

- Maximum height of the tree (Hmax): We collected the total height with the aid of a tape measure.

- Stem-specific density (SSD): We collected a piece of sapwood, took it to the laboratory, and removed the bark (suber), then the length and four diameters (two at the end) were measured with a pachymeter. The samples were dried in an oven at 65 °C for 72 h, after which their dry weight was measured.

- Relative bark thickness (weighted by trunk diameter at breast height) (BT): We used a chisel to remove the suber, took four measurements per individual, and used the diameters from the inventory for standardization.

- Leaf thickness (LT): We took two measurements per leaf using a micrometer.

- Leaf area (LA): We collected three to five leaves per individual; then, we scanned all the leaves and filled in the parts with herbivory in GIMP 2.10.22, to calculate the leaf area in R program, using the *EBImage* package.

- Specific leaf area (SLA): We dried the same leaves used for calculated the leaf area in an oven at 65 °C for 72 h, then we took the dry weight and calculated the SLA in R.

- Leaf concentrations of phosphorus (P), potassium (K), nitrogen (N), carbon (C), calcium (Ca), and magnesium (Mg): We collected three individuals per species, dried them in an oven at 65 °C for 72 h, and then sent them to the laboratory to determine the concentration of each leaf nutrient.

**Additional result**: For 32 species in riparian forest in forested catchments and 35 in riparian forest in cropland catchments, we found fewer than three individuals per species.

**Additional methods**: we made the map in QGIS software, version 3.30 (https://www.qgis.org/pt_BR/site/forusers/download.html), with the aid of the Globo Builder and QuickMapServices add-ons using Google Satellite images. This map was elaborated by the co-authors Silva and Silvério together with the main author Maracahipes-Santos.

We performed a reliability test of the results using random subsamples of these data (bootstrapping at 95%).


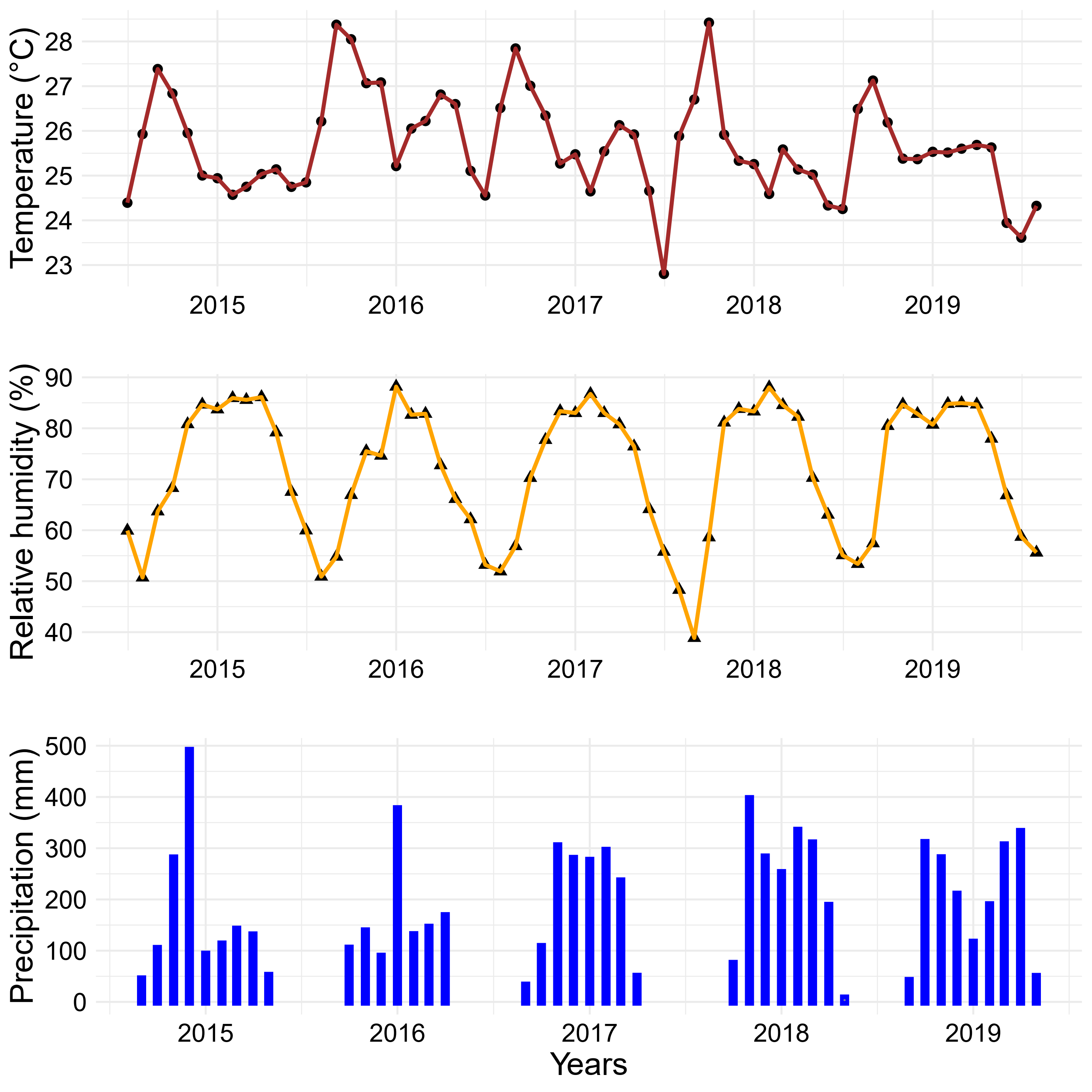
 **Fig. S1**. Average air temperature, relative humidity, and precipitation measured at the central weather Station of the Instituto de Pesquisa Ambiental da Amazônia, located at Fazenda Tanguro, Querência-MT, Brazil.


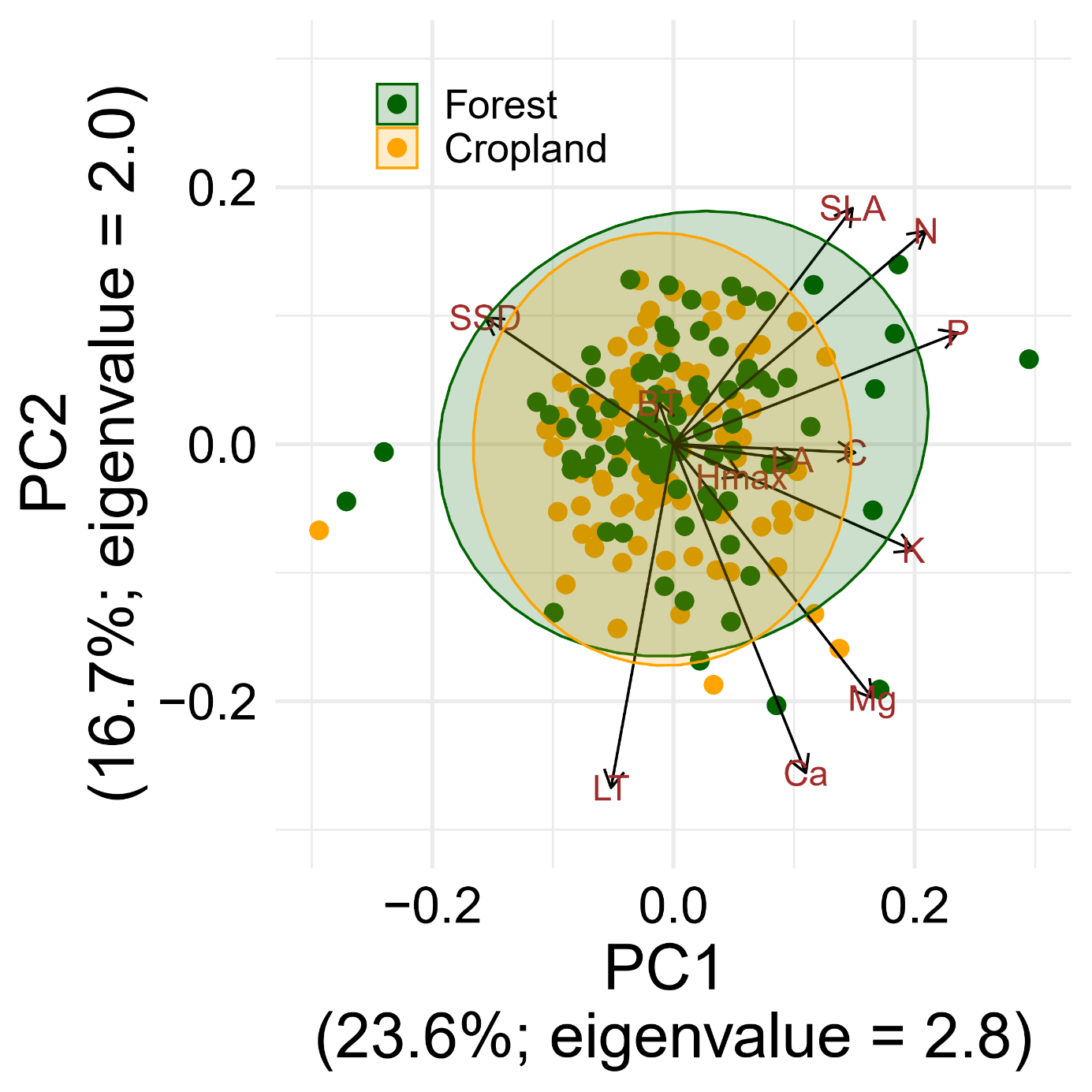


**Figure S2.** Principal Component Analysis of functional traits of tree species riparian forests in forested catchments (Forest) and riparian forests in cropland catchments (Cropland) in the southern Amazonia, Querência–MT, Brazil. Hmax = maximum tree height; BT = bark thickness; SSD = stem-specific density; LA = leaf area; LT = leaf thickness, SLA = specific leaf area, C = leaf carbon concentration, N = leaf nitrogen concentration, K = leaf potassium concentration, P = leaf phosphorus concentration, Ca = leaf calcium concentration, and Mg = leaf magnesium concentration. Forest and cropland catchments differed significantly (MANOVA: F_(1, 184)_ = 4.36, p = 0.038).


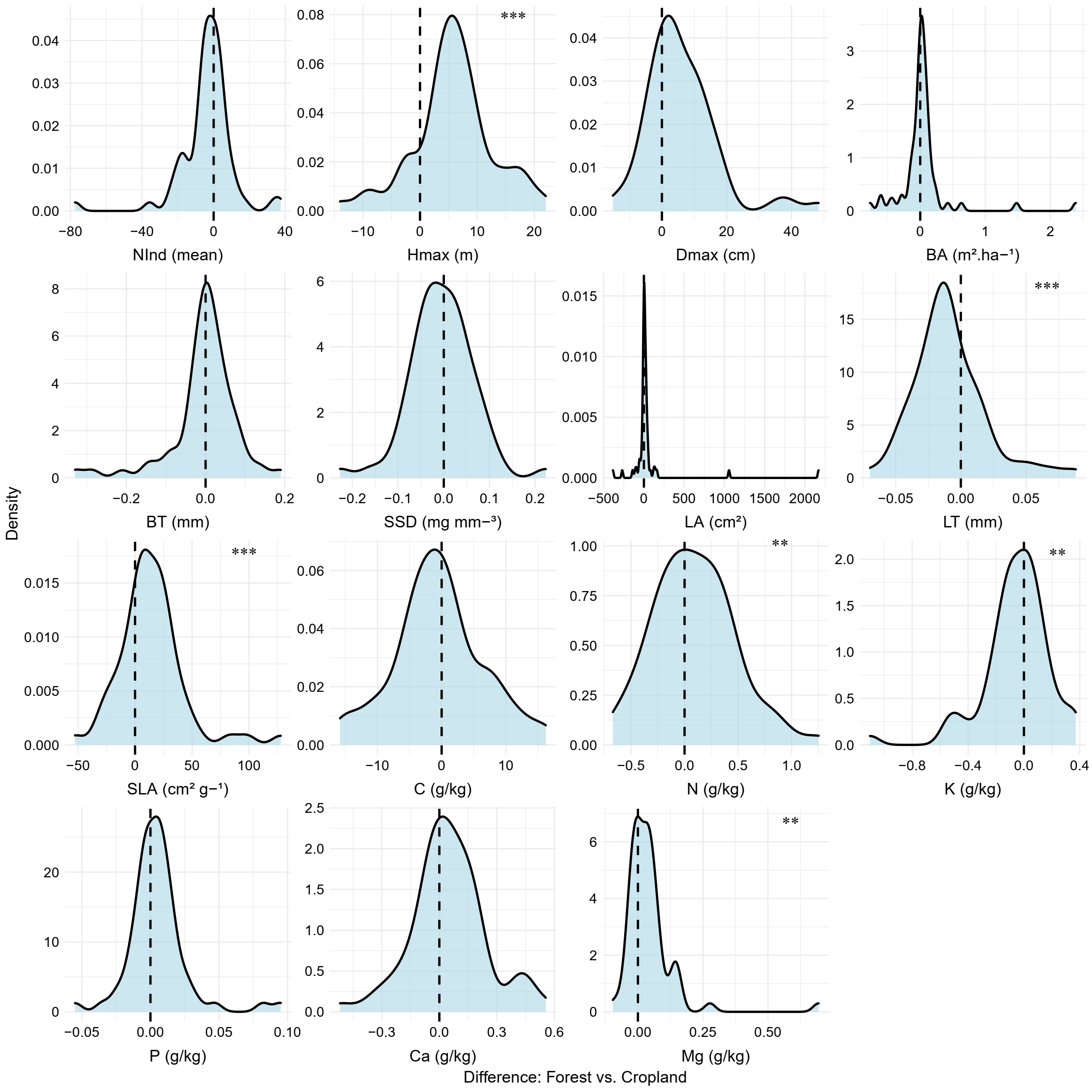


**Fig. S3**. Difference for 15 functional traits of tree species (abundance-weighted average) of the riparian forests in forested catchments (Forest) and riparian forests in cropland catchments (Croplands) in the southern Amazonia, Querência–MT, Brazil. NInd = mean number of individuals per species; Hmax = maximum tree height; Dmax = maximum diameter per species; BA = mean basal area per species; BT = bark thickness; SSD = stem-specific density; LA = leaf area; LT = leaf thickness, SLA = specific leaf area, C = leaf carbon concentration, N = leaf nitrogen concentration, K = leaf potassium concentration, P = leaf phosphorus concentration, Ca = leaf calcium concentration, and Mg = leaf magnesium concentration. We use a 5% significance level in this analysis. Positive values indicate that forested catchments had greater values than cropland catchments.


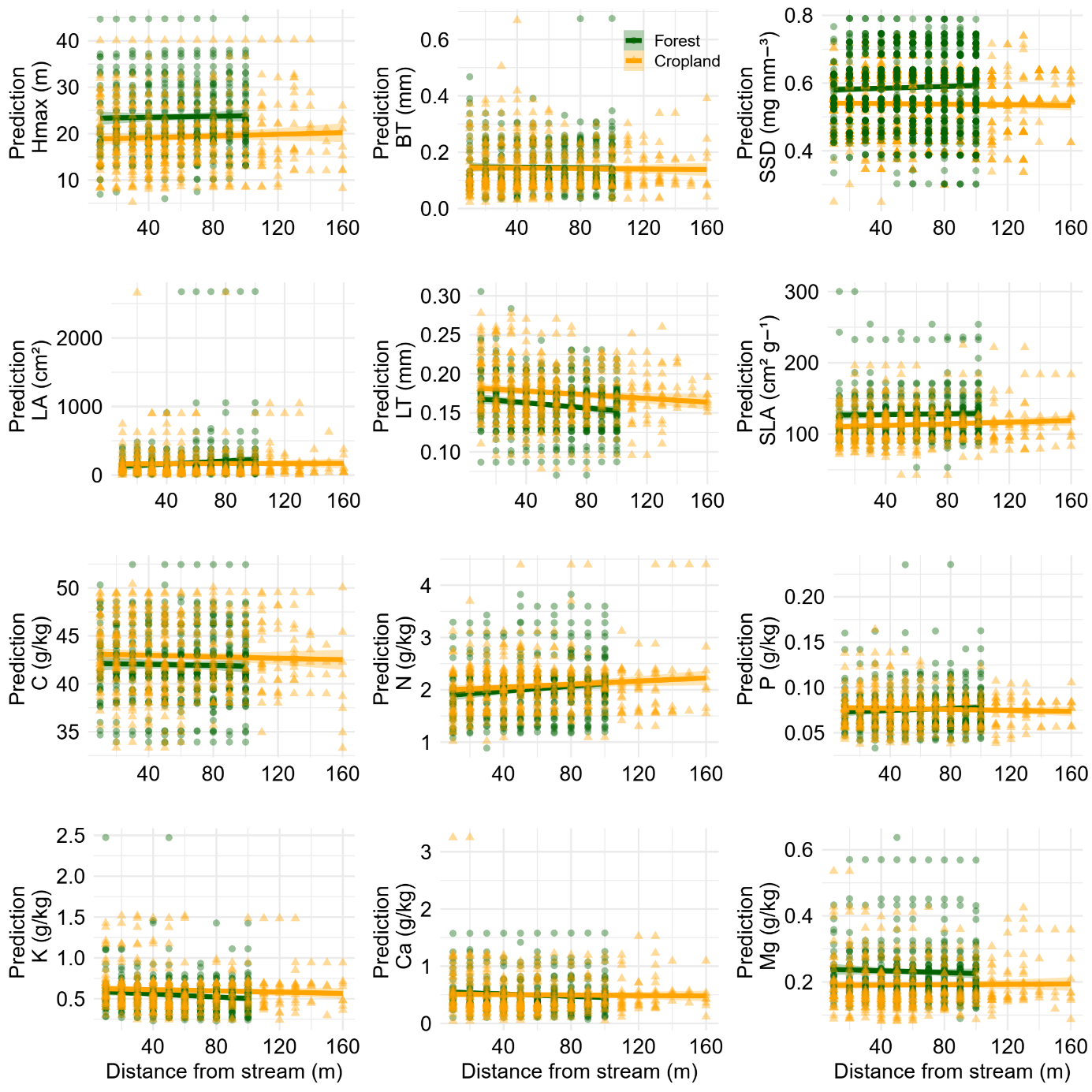
**Fig. S4**. Generalized Linear Mixed Model fitted for functional traits of tree species representing a sampling gradient from stream (0 m) to edge (40 to 160 m) (riparian forests in cropland catchments; Croplands) and from stream (0 m) to the greatest distance sampled from the edge (100 m) (riparian forests in forested catchments; Forest) in riparian forests in southern Amazonia, Querência–MT, Brazil. Hmax = maximum tree height; BT = bark thickness; SSD = stem-specific density; LA = leaf area; LT = leaf thickness, SLA = specific leaf area, C = leaf carbon concentration, N = leaf nitrogen concentration, K = leaf potassium concentration, P = leaf phosphorus concentration, Ca = leaf calcium concentration, and Mg = leaf magnesium concentration. We use a 5% significance level in this analysis.


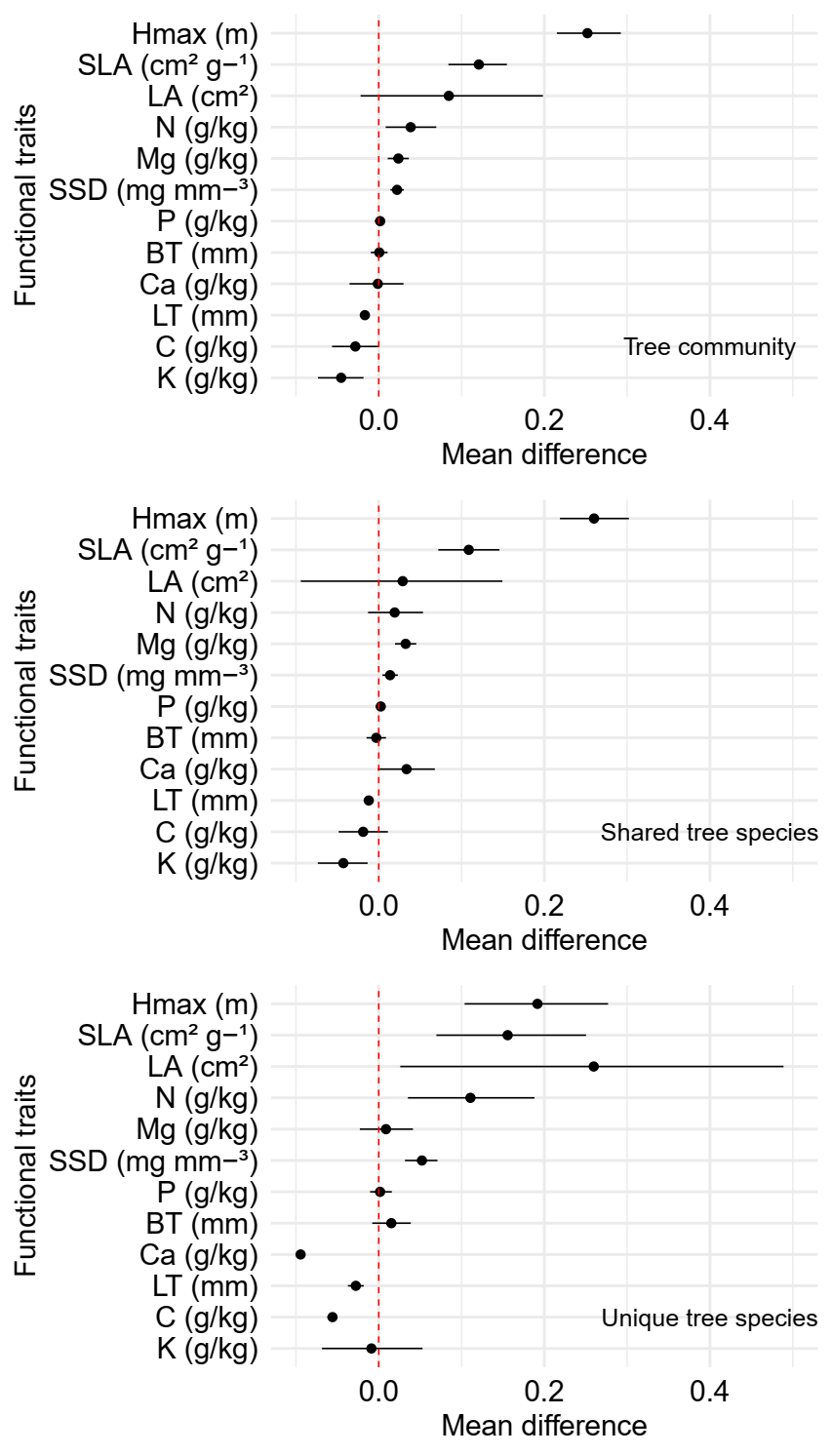


**Fig. S5**. Reliability analysis (bootstrapping at 95%) for tree community, shared species and excluded species from riparian forests in forested (Forest) and cropland (Cropland) catchments in southern Amazonia, Querência–MT, Brazil. Hmax = maximum tree height; BT = bark thickness; SSD = stem-specific density; LA = leaf area; LT = leaf thickness, SLA = specific leaf area, C = leaf carbon concentration, N = leaf nitrogen concentration, K = leaf potassium concentration, P = leaf phosphorus concentration, Ca = leaf calcium concentration, and Mg = leaf magnesium concentration.

**Table S1**. Functional significance, prediction, and theoretical rationale for tree functional traits in riparian forests in the southern Amazonia, Querência, MT, Brazil. Forest = riparian forests in forested catchments; cropland = riparian forests in cropland catchments.

| **Functional Traits** | **Code** | **Unit** | **Functional Significance** | **Prediction** | | | **Foundation** |
| --- | --- | --- | --- | --- | --- | --- | --- |
|  |  |  |  | **“Forest”** | **“Cropland”** |  | |
| Maximum tree height | Hmax | m | Tolerance or resistance to disturbances (e.g., fire, storm, tillage and grazing), total fertilization and competitive ability of trees^1^. | High | Low | Trees in forested catchments invest in height, due to greater competition for light in a more closed canopy. The edge effect also reduces the height of species through various physical disturbances (e.g., harsher winds). | |
| Stem-specific density | SSD | mg mm^-3^ | Resistance to physical damage and structural support of the tree^1–3^. | High | Low | Higher stem-specific density in forested catchments, as forests in cropland catchments show a change in species composition with the presence of pioneer species that have low wood density. Forest trees are taller and need more physical strength, whereas forests in cropland catchments are drier and storing water in the trunk can be a good strategy. | |
| Bark thickness standardized by diameter | BT | mm | Protection of tree tissues from damage, mainly related to fire disturbance^1^. | Minor | Major | Adaptation of forest species in cropland catchments to eventual fire events with increased bark thickness. Forests in cropland catchments are drier with more grasses and therefore more prone to burning. | |
| Leaf thickness | LT | mm | Protection from physical damage and relation to the tree's strategy for resource acquisition and use^4,5^. | Minor | Major | Adaptation of forest species in cropland catchments to withstand increased light, increased radiation and temperature, differences in exposure and wind dynamics^6^. Why do trees in forested catchments invest in thinner leaves to better capture the limiting light. | |
| Leaf area | LA | cm^2^ | Shows responses to environmental stresses and disturbances, with respect to resource acquisition and use. With a tendency to select small leaves in the presence of environmental stresses and disturbances^1^. | High | Low | Species from forested catchments tend to have a larger average leaf area, due to the fact that light is a limiting resource within the forest, and this allows for greater light absorption by the leaves. | |
| Specific leaf area | SLA | cm^2^ g^-1^ | Related to resource acquisition, resource use efficiency, structural defense, and tree growth strategy. It also shows a negative correlation with the life span of leaves^1^. | High | Low | Species in forested catchments with higher nutrient availability can invest in a larger leaf area per unit leaf mass, this increases growth and improves the species' ability to compete for light within the forest. | |
| Leaf carbon concentration | C | g/kg | Lower leaf carbon concentration suggests higher nutritional quality and more palatable leaves^1^. | Low | High | Species in cropland catchments are susceptible to abiotic (e.g., temperature, fire increase in groundwater etc.) and biotic stress (e.g., microorganisms and grasses etc.) due to land use changes. This promotes increased leaf toughness and influences leaf protection through carbon assimilation. | |
| Leaf nitrogen concentration | N | g/kg | Nitrogen is a nutrient that influences the photosynthetic capacity and growth of trees. It is an important limiting macronutrient in trees^7,8^. | High | Low | Trees species in forested catchments with nutrient-rich soils have higher leaf nitrogen concentrations and photosynthetic rates than species in riparian forests in cropland catchments with poorer soils. Because of competition for light in forested catchments. | |
| Leaf phosphorus concentration | P | g/kg | Nutrient influencing tree growth and productivity^9^. | High | Low | Soils in forested catchments have higher concentrations of this nutrient than in forests in cropland catchments. In addition, forested catchments are more efficient at cycling nutrients via burlap. Why are forested catchments more efficient at nutrient cycling via burlap, whereas forests in cropland catchments have their nutrient cycling dynamics compromised due to changes in microclimate, species composition, deposition of allochthonous material from the disturbed landscape. | |
| Leaf potassium concentration | K | g/kg | It plays a key role in tree growth and metabolism and helps the survival of trees exposed to various biotic and abiotic stresses^10^. | High | Low | Similar rationale as for phosphorus. | |
| Leaf calcium concentration | Ca | g/kg | It plays essential physiological roles in tree structure, with increased strength, and contributes to healthy leaf growth^8,11^. | High | Low | Similar rationale as for phosphorus. | |
| Leaf magnesium concentration | Mg | g/kg | Contributes to the photosynthetic process of trees and is related to tree growth and health^12^. | High | Low | Similar rationale as for phosphorus. | |

**Table S2**. List of riparian forest tree species from which we collected functional traits in southern Amazonia, Querência–MT, Brazil. Forest = riparian forests in forested catchments; Cropland = riparian forests in cropland catchments. We sort species by botanical families.

| **Species/Family** | **Forest** | **Cropland** |
| --- | --- | --- |
| **Anacardiaceae** |  |  |
| 1. *Tapirira guianensis* Aubl. | × | × |
| 2. *Tapirira obtusa* (Benth.) J.D.Mitch. | × | – |
| **Annonaceae** |  |  |
| 3. *Bocageopsis mattogrossensis* (R.E.Fr.) R.E.Fr. | × | × |
| 4. *Xylopia amazonica* R.E.Fr. | × | × |
| 5. *Guatteria schomburgkiana* Mart. | × | × |
| 6. *Annona insignis* R.E.Fr. | × | × |
| 7. *Guatteria blepharophylla* Mart. | × | × |
| 8. *Xylopia* sp. 1 | × | × |
| 9. *Xylopia* sp. 2 | × | × |
| **Apocynaceae** |  |  |
| 10. *Aspidosperma excelsum* Benth. | × | × |
| 11. *Himatanthus bracteatus* (A. DC.) Woodson | × | – |
| 12. *Aspidosperma desmanthum* Benth. ex Müll.Arg. | × | – |
| **Araliaceae** |  |  |
| 13. *Didymopanax morototoni* (Aubl.) Decne. & Planch. | × | × |
| **Bignoniaceae** |  |  |
| 14. *Jacaranda copaia* (Aubl.) D.Don | × | × |
| **Boraginaceae** |  |  |
| 15. *Cordia bicolor* A.DC. | × | × |
| **Burseraceae** |  |  |
| 16. *Protium pilosissimum* Engl. | × | × |
| 17. *Protium spruceanum* (Benth.) Engl. | × | × |
| 18. *Trattinnickia glaziovii* Swart | × | × |
| 19. *Protium unifoliolatum* Engl. | × | × |
| 20. *Trattinnickia burserifolia* Mart. | × | × |
| 21. *Dacryodes microcarpa* Cuatrec. | × | – |
| 22. *Trattinnickia rhoifolia* Willd. | × | × |
| **Calophyllaceae** |  |  |
| 23. *Calophyllum brasiliense* Cambess. | – | × |
| **Chrysobalanaceae** |  |  |
| 24. *Hirtella bicornis* Mart. & Zucc. | × | – |
| 25. *Licania longistyla* (Hook.f.) Fritsch | × | × |
| 26. *Licania egleri* Prance | × | × |
| 27. *Hirtella glandulosa* Spreng. | – | × |
| **Clusiaceae** |  |  |
| 28. *Garcinia madruno* (Kunth) Hammel | × | – |
| 29. *Symphonia globulifera* L.f. | – | × |
| **Combretaceae** |  |  |
| 30. *Buchenavia tetraphylla* (Aubl.) R.A. Howard | × | × |
| **Connaraceae** |  |  |
| 31. *Connarus perrottetii* (DC.) Planch. | × | × |
| **Ebenaceae** |  |  |
| 32. *Diospyros sericea* A.DC. | – | × |
| **Elaeocarpaceae** |  |  |
| 33. *Sloanea sinemariensis* Aubl. | × | × |
| 34. *Sloanea erismoides* Ducke | × | × |
| **Erythroxylaceae** |  |  |
| 35. *Erythroxylum rufum* Cav. | × | – |
| **Euphorbiaceae** |  |  |
| 36. *Aparisthmium cordatum* (A.Juss.) Baill. | × | – |
| 37. *Maprounea guianensis* Aubl. | × | × |
| 38. *Alchornea discolor* Poepp. | – | × |
| 39. *Mabea fistulifera* Mart. | – | × |
| **Fabaceae** |  |  |
| 40. *Diplotropis purpurea* (Rich.) Amshoff | × | – |
| 41. *Inga thibaudiana* DC. | × | × |
| 42. *Ormosia paraensis* Ducke | × | × |
| 43. *Inga heterophylla* Willd. | × | × |
| 44. *Apuleia leiocarpa* (Vogel) J.F.Macbr. | × | – |
| 45. *Enterolobium schomburgkii* (Benth.) Benth. | × | × |
| 46. *Hymenaea courbaril* L. | × | × |
| 47. *Inga alba* (Sw.) Willd. | – | × |
| 48. *Bauhinia dubia* G.Don | – | × |
| 49. *Tachigali vulgaris* L.G.Silva & H.C.Lima | – | × |
| **Humiriaceae** |  |  |
| 50. *Sacoglottis guianensis* Benth. | × | × |
| **Hypericaceae** |  |  |
| 51. *Vismia japurensis* Reichardt | – | × |
| **Lauraceae** |  |  |
| 52. *Ocotea guianensis* Aubl. | × | – |
| 53. *Ocotea leucoxylon* (Sw.) Laness. | × | × |
| 54. *Nectandra cuspidata* Nees | × | × |
| 55. *Mezilaurus* sp. | × | × |
| 56. *Ocotea aciphylla* (Nees & Mart.) Mez | × |  |
| 57. *Ocotea cujumary* Mart. | × | × |
| 58. *Nectandra cissiflora* Nees | – | × |
| **Lythraceae** |  |  |
| 59. *Physocalymma scaberrimum* Pohl | × | × |
| **Magnoliaceae** |  |  |
| 60. *Magnolia ovata* (A.St.-Hil.) Spreng. | – | × |
| **Malpighiaceae** |  |  |
| 61. *Byrsonima aerugo* Sagot | × | × |
| **Malvaceae** |  |  |
| 62. *Luehea* sp. | × | × |
| 63. *Mollia lepidota* Spruce ex Benth. | × | × |
| 64. *Eriotheca surinamensis* (Uittien) A.Robyns | × | – |
| 65. *Eriotheca globosa* (Aubl.) A.Robyns | – | × |
| **Melastomataceae** |  |  |
| 66. *Miconia punctata* (Desr.) DC. | × | – |
| 67. *Miconia pyrifolia* Naudin | × | – |
| 68. *Miconia* sp. | × | × |
| 69. *Mouriri brachyanthera* Ducke | × | – |
| 70. *Miconia biglandulosa* Gleason | × | × |
| 71. *Miconia holosericea* (L.) DC. | × | × |
| 72. *Miconia minutiflora* (Bonpl.) DC. | × | × |
| 73. *Miconia prasina* (Sw.) DC. | × | – |
| 74. *Miconia gratissima* Benth. ex Triana | × | × |
| 75. *Bellucia grossularioides* (L.) Triana | × | × |
| **Meliaceae** |  |  |
| 76. *Guarea guidonia* (L.) Sleumer | × | × |
| 77. *Trichilia quadrijuga* Kunth | – | × |
| **Moraceae** |  |  |
| 78. *Pseudolmedia macrophylla* Trécul | × | – |
| 79. *Ficus mathewsii* (Miq.) Miq. | × | × |
| 80. *Ficus matiziana* Dugand | – | × |
| 81. *Ficus maxima* Mill. | – | × |
| 82. *Sorocea guilleminiana* Gaudich. | – | × |
| **Myristicaeae** |  |  |
| 83. *Virola sebifera* Aubl. | × | × |
| 84. *Virola malmei* A.C.Sm. | – | × |
| **Myrtaceae** |  |  |
| 85. *Myrcia multiflora* (Lam.) DC. | × | × |
| 86. *Myrcia umbraticola* (O.Berg) E.Lucas | × | × |
| 87. *Myrcia splendens* (Sw.) DC. | × | × |
| 88. *Myrcia eximia* DC. | × | × |
| 89. *Myrcia sylvatica* (G.Mey.) DC. | – | × |
| **Ochnaceae** |  |  |
| 90. *Ouratea discophora* Ducke | × | × |
| **Peraceae** |  |  |
| 91. *Pera glabrata* (Schott) Baill. | – | × |
| 92. *Chaetocarpus schomburgkianus* (Kuntze) Pax & K.Hoffm. | × | × |
| **Phyllanthaceae** |  |  |
| 93. *Hyeronima* *alchorneoides* Allemão | × | × |
| **Quiinaceae** |  |  |
| 94. *Quiina florida* Tul. | – | × |
| **Rubiaceae** |  |  |
| 95. *Amaioua guianensis* Aubl. | × | × |
| 96. *Ferdinandusa guainiae* Spruce ex K.Schum. | × | × |
| **Salicaceae** |  |  |
| 97. *Casearia grandiflora* Cambess. | – | × |
| **Sapindaceae** |  |  |
| 98. *Matayba inelegans* Spruce ex Radlk. | × | – |
| 99. *Matayba arborescens* (Aubl.) Radlk. | × | – |
| 100. *Talisia cerasina* (Benth.) Radlk. | × | – |
| **Sapotaceae** |  |  |
| 101. *Micropholis egensis* (A.DC.) Pierre | × | × |
| 102. *Pouteria ramiflora* (Mart.) Radlk. | × | × |
| 103. *Pouteria cuspidata* (A.DC.) Baehni | × | × |
| **Simaroubaceae** |  |  |
| 104. *Simarouba amara* Aubl. | – | × |
| **Siparunaceae** |  |  |
| 105. *Siparuna guianensis* Aubl. | – | × |
| **Urticaceae** |  |  |
| 106. *Cecropia pachystachya* Trécul | – | × |
| 107. *Cecropia distachya* Huber | – | × |
| 108. *Pourouma velutina* Mart. ex Miq. | – | × |
| **Vochysiaceae** |  |  |
| 109. *Ruizterania wittrockii* (Malme) Marc.-Berti | × | × |
| 110. *Vochysia vismiifolia* Spruce ex Warm. | × | × |
| 111. *Qualea ingens* Warm. | × | × |
| 112. *Vochysia ferruginea* Mart. | – | × |
| **Unidentified** |  |  |
| 113. NI. 7 | × | – |
| 114. NI. 8 | × | – |
| 115. NI. 12 | × | – |
| 116. NI. 13 | × | – |
| 117. NI. 1 | – | × |
| 118. NI. 2 | – | × |
| 119. NI. 9 | – | × |
| 120. NI. 10 | – | × |
| 121. NI. 11 | – | × |
| 122. NI. 14 | × | × |
| 123. NI. 6 | × | – |

**Table S3.** Multicollinearity among functional traits of riparian forests in the southern Amazonia, Querência–MT, Brazil. VIF = Variance Inflation Factor.

| **Functional Traits** | **Unit** | **Symbols** | **VIF** |
| --- | --- | --- | --- |
| Maximum tree height | m | Hmax | 1.09 |
| Stem-specific density | mg mm^-3^ | SSD | 1.54 |
| Bark thickness | mm | BT | 1.08 |
| Leaf thickness | mm | LT | 1.53 |
| Leaf area | cm^2^ | LA | 1.35 |
| Specific leaf area | cm^2^ g^-1^ | SLA | 1.59 |
| Leaf carbon concentration | g/kg | C | 1.29 |
| Leaf nitrogen concentration | g/kg | N | 1.97 |
| Leaf phosphorus concentration | g/kg | P | 2.07 |
| Leaf potassium concentration | g/kg | K | 1.58 |
| Leaf calcium concentration | g/kg | Ca | 1.86 |
| Leaf magnesium concentration | g/kg | Mg | 1.87 |

**Table S4**. Multivariate Analysis of Variance between functional traits of tree species from riparian forests in forested catchments (Forest) and riparian forests in cropland catchments (Croplands) in the southern Amazonia, Querência–MT, Brazil. Ecological strategies of tree species in riparian forests (F _(1, 323)_ = 24.12; p < 0.001). Degrees of freedom for all functional traits (df = 1, 440).

| **Functional Traits** | **Unit** | **Symbols** | **Average (SD)** | | **F** | **p** | |
| --- | --- | --- | --- | --- | --- | --- | --- |
|  |  |  | Forest | Cropland |  |  |  |
| Maximum tree height | m | Hmax | 23.19 (7.84) | 18.08 (7.34) | 43.38 | < **0.001** |  |
| Stem-specific density | mg mm^-3^ | SSD | 0.59 (0.13) | 0.55 (0.11) | 0.12 | 0.731 |  |
| Bark thickness | mm | BT | 0.15 (0.12) | 0.15 (0.13) | 0.06 | 0.810 |  |
| Leaf thickness | mm | LT | 0.16 (0.04) | 0.18 (0.05) | 26.89 | < **0.001** |  |
| Leaf area | cm^2^ | LA | 149.37 (274.44) | 162.82 (263.70) | 0.39 | 0.532 |  |
| Specific leaf area | cm^2^ g^-1^ | SLA | 127.15 (51.04) | 112.32 (38.05) | 36.37 | < **0.001** |  |
| Leaf carbon concentration | g/kg | C | 41.78 (7.24) | 42.77 (7.66) | 3.14 | 0.077 |  |
| Leaf nitrogen concentration | g/kg | N | 2.06 (0.62) | 1.95 (0.63) | 5.56 | **0.019** |  |
| Leaf phosphorus concentration | g/kg | P | 0.08 (0.03) | 0.08 (0.03) | 0.10 | 0.747 |  |
| Leaf potassium concentration | g/kg | K | 0.54 (0.28) | 0.63 (0.35) | 6.59 | **0.011** |  |
| Leaf calcium concentration | g/kg | Ca | 0.49 (0.36) | 0.50 (0.42) | 0.04 | 0.844 |  |
| Leaf magnesium concentration | g/kg | Mg | 0.23 (0.12) | 0.20 (0.09) | 10.49 | **0.001** |  |

**Table S5.** Effect of turnover on tree species composition, intraspecific trait variability, and their covariation between riparian forests in forested catchments (Forest) and riparian forests in cropland catchments (Croplands) in the southern Amazonia, Fazenda Tanguro, Querência–MT, Brazil. Hmax = Maximum tree height (m); SSD = Stem-specific density (mg mm^-3^); BT = Bark thickness (mm); LT = Leaf thickness (mm); LA = Leaf area (cm^2^); SLA = Specific leaf area (cm^2^ g^-1^); C = Leaf carbon concentration (g/kg); N = Leaf nitrogen concentration (g/kg); P = Leaf phosphorus concentration (g/kg); K = Leaf potassium concentration (g/kg); Ca = Leaf calcium concentration (g/kg); Mg = Leaf magnesium concentration (g/kg). ^#^ = Total – specific average.

| **Functional traits** | **Effects** | **Turnover** | **Intraspecific** | **Covariation** | **Total ^#^** |
| --- | --- | --- | --- | --- | --- |
| Hmax (m) | Total | 0.25 | 0.57 | 0.18 | 1.00 |
|  | Residuals | 0.17 | 0.33 | -0.09 | 0.41 |
|  | P | 0.10 | **0.04** | - | **0.01** |
| SSD (mg mm^-3^) | Total | 0.47 | 0.46 | 0.07 | 1.00 |
|  | Residuals | 0.25 | 0.32 | -0.29 | 0.28 |
|  | P | **0.03** | 0.09 | - | **0.00** |
| BT (mm) * | Total | 0.98 | 0.99 | 0.97 | 1.00 |
|  | Residuals | 0.98 | 0.87 | -0.96 | 0.89 |
|  | P | 0.97 | 0.32 | - | 0.34 |
| LT (mm) | Total | 1.19 | 1.00 | -1.19 | 1.00 |
|  | Residuals | 0.85 | 0.97 | -1.42 | 0.40 |
|  | P | 0.11 | 0.58 | - | **0.01** |
| LA (cm^2^) | Total | 0.87 | 0.52 | -0.40 | 1.00 |
|  | Residuals | 0.78 | 0.50 | -0.50 | 0.78 |
|  | P | 0.35 | 0.54 | - | 0.17 |
| SLA (cm^2^ g^-1^) | Total | 0.40 | 0.46 | 0.14 | 1.00 |
|  | Residuals | 0.28 | 0.30 | -0.13 | 0.45 |
|  | P | 0.10 | 0.08 | - | **0.01** |
| C (g/kg) | Total | 0.55 | 1.07 | -0.62 | 1.00 |
|  | Residuals | 0.55 | 0.84 | -0.67 | 0.72 |
|  | P | 0.85 | 0.18 | - | 0.11 |
| N (g/kg) | Total | 0.84 | 0.34 | -0.18 | 1.00 |
|  | Residuals | 0.83 | 0.05 | -0.09 | 0.79 |
|  | P | 0.80 | **0.00** | - | 0.18 |
| P (g/kg) | Total | 2.53 | 1.85 | -3.38 | 1.00 |
|  | Residuals | 2.22 | 1.81 | -3.60 | 0.44 |
|  | P | 0.33 | 0.69 | - | **0.01** |
| K (g/kg) | Total | 0.84 | 0.76 | -0.60 | 1.00 |
|  | Residuals | 0.81 | 0.49 | -0.77 | 0.53 |
|  | P | 0.62 | 0.07 | - | **0.03** |
| Ca (g/kg) | Total | 1.55 | 0.62 | -1.17 | 1.00 |
|  | Residuals | 1.47 | 0.38 | -0.89 | 0.96 |
|  | P | 0.53 | 0.05 | - | 0.56 |
| Mg (g/kg) | Total | 0.43 | 0.87 | -0.31 | 1.00 |
|  | Residuals | 0.43 | 0.21 | -0.39 | 0.25 |
|  | P | 0.84 | **0.00** | - | **0.00** |

**Table S6**. Multivariate Analysis of Variance between functional traits of tree species from riparian forests in forested catchments (Forest) and riparian forests in cropland catchments (Croplands) in the southern Amazonia, Querência–MT, Brazil. Variability of functional traits of shared tree species in the two riparian forest environments (F _(1, 265)_ = 20.51; p < 0.001). Degrees of freedom for all functional traits (df = 1, 265).

| **Functional Traits** | **Unit** | **Symbols** | **Average (SD)** | | **F** | **p** |
| --- | --- | --- | --- | --- | --- | --- |
|  |  |  | Forest | Cropland |  |  |
| Maximum tree height | m | Hmax | 23.75 (7.96) | 18.42 (7.24) | 194.43 | < **0.001** |
| Stem-specific density | mg mm^-3^ | SSD | 0.58 (0.12) | 0.56 (0.11) | 0.60 | 0.438 |
| Bark thickness | mm | BT | 0.14 (0.11) | 0.15 (0.13) | 0.01 | 0.916 |
| Leaf thickness | mm | LT | 0.16 (0.04) | 0.17 (0.05) | 21.39 | < **0.001** |
| Leaf area | cm^2^ | LA | 157.35 (297.83) | 155.16 (220.66) | 3.92 | **0.049** |
| Specific leaf area | cm^2^ g^-1^ | SLA | 126.75 (42.63) | 113.27 (34.71) | 25.60 | < **0.001** |
| Leaf carbon concentration | g/kg | C | 41.92 (7.48) | 42.79 (7.90) | 0.87 | 0.351 |
| Leaf nitrogen concentration | g/kg | N | 2.01 (0.55) | 1.96 (0.59) | 2.51 | 0.115 |
| Leaf phosphorus concentration | g/kg | P | 0.08 (0.03) | 0.07 (0.02) | 4.35 | **0.038** |
| Leaf potassium concentration | g/kg | K | 0.51 (0.25) | 0.58 (0.33) | 6.79 | **0.010** |
| Leaf calcium concentration | g/kg | Ca | 0.50 (0.37) | 0.44 (0.29) | 1.86 | 0.277 |
| Leaf magnesium concentration | g/kg | Mg | 0.22 (0.12) | 0.18 (0.07) | 22.82 | < **0.001** |

**Table S7**. Multivariate Analysis of Variance between functional traits of tree species unique to riparian forests in forested catchments (Forest) and riparian forests in cropland catchments (Croplands) in the southern Amazonia, Querência–MT, Brazil. Ecological strategies of unique tree species among riparian forests (F _(1, 48)_ = 2.31; p < 0.026). Degrees of freedom for all functional traits (df = 1, 48).

| **Functional Traits** | **Unit** | **Symbols** | **Average (SD)** | | **F** | **p** |
| --- | --- | --- | --- | --- | --- | --- |
|  |  |  | Forest | Cropland |  |  |
| Maximum tree height | m | Hmax | 20.30 (6.58) | 16.98 (7.59) | 0.25 | 0.622 |
| Stem-specific density | mg mm^-3^ | SSD | 0.61 (0.15) | 0.52 (0.11) | 1.73 | 0.195 |
| Bark thickness | mm | BT | 0.16 (0.13) | 0.14 (0.13) | 0.64 | 0.429 |
| Leaf thickness | mm | LT | 0.17 (0.04) | 0.20 (0.06) | 1.45 | 0.235 |
| Leaf area | cm^2^ | LA | 114.02 (122.01) | 186.98 (368.18) | 1.24 | 0.272 |
| Specific leaf area | cm^2^ g^-1^ | SLA | 128.94 (78.42) | 109.31 (47.13) | 6.46 | **0.014** |
| Leaf carbon concentration | g/kg | C | 42.17 (6.11) | 42.70 (7.09) | 3.41 | 0.071 |
| Leaf nitrogen concentration | g/kg | N | 2.27 (0.82) | 1.92 (0.73) | 1.94 | 0.170 |
| Leaf phosphorus concentration | g/kg | P | 0.08 (0.05) | 0.08 (0.03) | 0.02 | 0.889 |
| Leaf potassium concentration | g/kg | K | 0.70 (0.36) | 0.73 (0.38) | 0.17 | 0.680 |
| Leaf calcium concentration | g/kg | Ca | 0.46 (0.31) | 0.64 (0.61) | 1.40 | 0.243 |
| Leaf magnesium concentration | g/kg | Mg | 0.24 (0.14) | 0.23 (0.13) | 0.86 | 0.358 |

**Table S8.** Summaries of Linear Mixed Effects Models between each functional trait of riparian forests in forested catchments (Forest) and riparian forests in cropland catchments (Croplands) in the southern Amazonia, Fazenda Tanguro, Querência–MT, Brazil.

| **Functional Traits** | **Fixed effects** | **Symbols** | **Estimate** | **Standard Error** | **df** | **t** | **p** |
| --- | --- | --- | --- | --- | --- | --- | --- |
| Maximum tree height (m) | Intercept | Hmax | 21.690 | 0.696 | 121.20 | 31.17 | **< 0.001** |
|  | Distance from the stream |  | 0.002 | 0.001 | 2483.00 | 1.17 | 0.243 |
|  | Land use |  | -4.634 | 0.099 | 2490.00 | -46.90 | **< 0.001** |
| Stem-specific density (mg mm^-3^) | Intercept | SSD | 0.562 | 0.010 | 119.40 | 55.55 | **< 0.001** |
|  | Distance from the stream |  | 0.000 | 0.000 | 2479.00 | -2.13 | **0.033** |
|  | Land use |  | -0.003 | 0.001 | 2482.00 | -3.17 | **0.002** |
| Bark thickness (mm) | Intercept | BT | 0.156 | 0.010 | 119.30 | 15.26 | **< 0.001** |
|  | Distance from the stream |  | 0.000 | 0.000 | 2480.00 | 2.55 | **0.011** |
|  | Land use |  | -0.004 | 0.001 | 2485.00 | -3.20 | **0.001** |
| Leaf thickness (mm) | Intercept | LT | 0.172 | 0.004 | 118.50 | 41.86 | **< 0.001** |
|  | Distance from the stream |  | 0.000 | 0.000 | 2478.00 | 0.98 | 0.327 |
|  | Land use |  | 0.008 | 0.000 | 2481.00 | 21.40 | **< 0.001** |
| Leaf area (cm^2^) | Intercept | LA | 170.833 | 27.812 | 123.64 | 6.14 | **< 0.001** |
|  | Distance from the stream |  | 0.063 | 0.054 | 2486.07 | 1.17 | 0.242 |
|  | Land use |  | -12.511 | 4.122 | 2492.93 | -3.04 | **0.002** |
| Specific leaf area (cm^2^ g^-1^) | Intercept | SLA | 127.100 | 3.589 | 119.70 | 35.42 | **< 0.001** |
|  | Distance from the stream |  | -0.004 | 0.005 | 2480.00 | -0.77 | 0.444 |
|  | Land use |  | -10.720 | 0.388 | 2484.00 | -27.66 | **< 0.001** |
| Leaf carbon concentration (g/kg) | Intercept | C | 41.600 | 0.411 | 131.10 | 101.13 | **< 0.001** |
|  | Distance from the stream |  | 0.000 | 0.001 | 2506.00 | -0.12 | 0.908 |
|  | Land use |  | 1.012 | 0.113 | 2528.00 | 8.95 | **< 0.001** |
| Leaf nitrogen concentration (g/kg) | Intercept | N | 1.990 | 0.055 | 119.90 | 35.94 | **< 0.001** |
|  | Distance from the stream |  | 0.000 | 0.000 | 2480.00 | 0.59 | 0.553 |
|  | Land use |  | 0.105 | 0.006 | 2484.00 | 17.32 | **< 0.001** |
| Leaf phosphorus concentration (g/kg) | Intercept | P | 0.081 | 0.003 | 118.80 | 30.06 | **< 0.001** |
|  | Distance from the stream |  | 0.000 | 0.000 | 2480.00 | 1.99 | **0.047** |
|  | Land use |  | -0.004 | 0.000 | 2484.00 | -11.85 | **< 0.001** |
| Leaf potassium concentration (g/kg) | Intercept | K | 0.607 | 0.029 | 119.40 | 21.26 | **< 0.001** |
|  | Distance from the stream |  | 0.000 | 0.000 | 2480.00 | -1.15 | 0.251 |
|  | Land use |  | 0.059 | 0.003 | 2485.00 | 17.54 | **< 0.001** |
| Leaf calcium concentration (g/kg) | Intercept | Ca | 0.553 | 0.037 | 118.60 | 15.11 | **< 0.001** |
|  | Distance from the stream |  | 0.000 | 0.000 | 2478.00 | 1.95 | 0.051 |
|  | Land use |  | -0.060 | 0.003 | 2481.00 | -17.51 | **< 0.001** |
| Leaf magnesium concentration (g/kg) | Intercept | Mg | 0.240 | 0.009 | 119.80 | 26.67 | **< 0.001** |
|  | Distance from the stream |  | 0.000 | 0.000 | 2482.00 | -1.29 | 0.198 |
|  | Land use |  | -0.040 | 0.001 | 2488.00 | -31.60 | **< 0.001** |

References

1. Pérez-Harguindeguy, N. *et al.* New handbook for standardised measurement of plant functional traits worldwide. *Aust J Bot* **61**, 167 (2013).

2. Chave, J. *et al.* Towards a worldwide wood economics spectrum. *Ecol Lett* **12**, 351–366 (2009).

3. Larjavaara, M. & Muller-Landau, H. C. Rethinking the value of high wood density. *Funct Ecol* **24**, 701–705 (2010).

4. Vile, D. *et al.* Specific leaf area and dry matter content estimate thickness in laminar leaves. *Ann Bot* **96**, 1129–1136 (2005).

5. Cianciaruso, M. v., Silva, I. A., Batalha, M. A., Gaston, K. J. & Petchey, O. L. The influence of fire on phylogenetic and functional structure of woody savannas: Moving from species to individuals. *Perspect Plant Ecol Evol Syst* **14**, 205–216 (2012).

6. Saunders, D. A., Hobbs, R. J. & Margules, C. R. Biological Consequences of Ecosystem Fragmentation: A Review. *Conservation Biology* **5**, 18–32 (1991).

7. Evans, J. R. Photosynthesis and nitrogen relationships in leaves of C3 plants. *Oecologia* **78**, 9–19 (1989).

8. Chapin, F. S., Matson, P. A. & Vitousek, P. M. *Principles of Terrestrial Ecosystem Ecology*. (Springer New York, 2011). doi:10.1007/978-1-4419-9504-9.

9. Schachtman, D. P., Reid, R. J. & Ayling, S. M. Phosphorus Uptake by Plants: From Soil to Cell. *Plant Physiol* **116**, 447–453 (1998).

10. Tripler, C. E., Kaushal, S. S., Likens, G. E. & Todd Walter, M. Patterns in potassium dynamics in forest ecosystems. *Ecol Lett* **9**, 451–466 (2006).

11. Gilliham, M. *et al.* Calcium delivery and storage in plant leaves: exploring the link with water flow. *J Exp Bot* **62**, 2233–2250 (2011).

12. Shaul, O. Magnesium transport and function in plants: the tip of the iceberg. *BioMetals* **15**, 307–321 (2002).
